# *N*‑Terminal Octylated Peptoid Hydrogels as 3D-Printable Cell Scaffolds and Proteolytically Robust Cargo Depots

**DOI:** 10.1021/acsnano.5c16998

**Published:** 2026-02-09

**Authors:** Il-Soo Park, Younghak Cho, Yen Jea Lee, Daniela Gutierrez, Ronald N. Zuckermann, Hyejeong Seong, Jae Hong Kim

**Affiliations:** † Electronic and Hybrid Materials Research Center, 58975 Korea Institute of Science and Technology, Seoul 02792, Republic of Korea; ‡ Brain Science Institute, Korea Institute of Science and Technology, Seoul 02792, Republic of Korea; § Materials Sciences Division, 1666 Lawrence Berkeley National Laboratory, Berkeley, California 94720, United States; ∥ Biomaterials Research Center, Korea Institute of Science and Technology, Seoul 02792, Republic of Korea; ⊥ Molecular Foundry, Lawrence Berkeley National Laboratory, Berkeley, California 94720, United States; # Division of Bio-Medical Science and Technology, KIST School, University of Science and Technology, Seoul 02792, Republic of Korea; ∇ Convergence Research Center for Solutions to Electromagnetic Interference in Future-Mobility, Korea Institute of Science and Technology, Seoul 02792, Republic of Korea; ○ GIST InnoCORE AI-Nano Convergence Institute for Early Detection of Neurodegenerative Diseases, Gwangju Institute of Science and Technology, Gwangju 61005, Republic of Korea

**Keywords:** peptoid, hydrogels, self-assembly, cell scaffolds, protease-resistant materials, cargo delivery

## Abstract

Supramolecular hydrogels that mimic the extracellular matrix (ECM) represent promising materials for tissue engineering and drug delivery. However, conventional hydrogels formed via the self-assembly of natural or synthetic building blocks often face a trade-off between biological functionality and biochemical stability, limiting their utility in long-term or protease-rich environments. Peptoids, a class of peptide-inspired, sequence-defined polymers, offer a compelling alternative due to their exceptional proteolytic resistance and bioactivity. Despite this potential, the development of supramolecular peptoid hydrogels has been hindered by the absence of backbone hydrogen bond donors, which limits long-range ordering necessary for efficient hydrogel formation. This work describes a short peptoid functionalized at the *N*-terminus with an octyl chain that readily self-assembles into hydrogels. Hydrophobic interactions among pendant octyl groups promote directional peptoid packing into highly ordered nanosheets, which interconnect to form a porous hydrogel network. These hydrogels exhibit tunable viscoelasticity, shear-thinning, and self-healing properties, enabling their use as inks for extrusion-based 3D printing. They support NIH-3T3 fibroblast adhesion, spreading, and proliferation, maintaining greater than 95% cell viability over 4 days. Moreover, the hydrogels retain their macroscopic integrity under protease-rich conditions, enabling sustained cargo release and uniform cellular uptake. Together, this study demonstrates a class of supramolecular peptoid hydrogelators that integrate biocompatibility, 3D printability, and proteolytic stability, providing a versatile platform for ECM-mimetic scaffolds in regenerative medicine and long-term therapeutic delivery.

## Graphical abstract

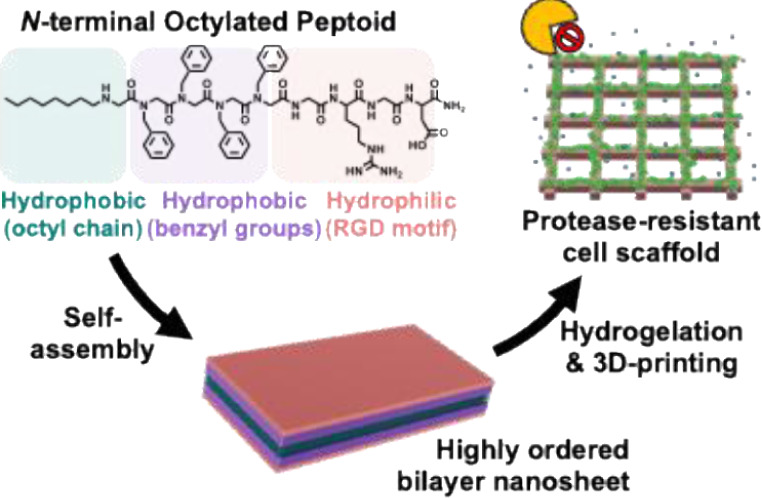

## 1 Introduction

Three-dimensional (3D) hydrated networks formed through the hierarchical organization of biopolymers represent fundamental structural motifs in biological systems. A notable example is the extracellular matrix (ECM), a dynamic scaffold primarily composed of proteoglycans and structural proteins such as elastin and collagen.^1,2^ The ECM consists of a fibrous 3D supramolecular network where reversible and irreversible interactions facilitate its hierarchical assembly.^3^ The dynamic nature of the ECM not only provides mechanical support, but also orchestrates essential cellular functions through the spatial presentation of biochemical cues that regulate cellular processes such as proliferation, differentiation, and intercellular communication.^4,5^ Therefore, mimicking the 3D architecture and dynamic multifunctionality of the ECM is crucial for controlling cell behavior and supporting tissue development. To emulate the multifunctionality of the ECM, covalently cross-linked hydrogels derived from natural or synthetic polymers have been extensively explored for biomedical applications, including tissue regeneration,^6,7^ drug delivery,^8^ and wound healing.^9,10^ While these covalently cross-linked networks offer mechanical robustness, they often lack dynamic mechanical behaviors (e.g., stress relaxation, shear-thinning, and self-healing), which are critical for emerging biomedical applications involving soft tissue integration, dynamic cell scaffolding, and 3D printing.^11^ To address these limitations, supramolecular hydrogels assembled via noncovalent interactions between building blocks have emerged as promising alternatives, offering tunable mechanical and dynamic properties. Peptide-based hydrogelators, in particular, provide advantages such as inherent bioactivity and design versatility.^12^ However, they are inherently susceptible to proteolytic degradation, leading to unpredictable structural disintegration, premature release of encapsulated cargos,^13^ and the generation of bioactive fragments that may elicit unintended biological responses.^14^ These challenges are particularly problematic in protease-rich environments such as those associated with chronic inflammation, autoimmune diseases, and tumor microenvironments.^15,16^ In contrast, synthetic building blocks typically exhibit robust proteolytic stability but often lack biological functionality. Bridging this trade-off requires the development of molecular building blocks that combine proteolytic stability with functional bioactivity, enabling supramolecular hydrogels that replicate ECM multifunctionality while maintaining structural integrity and predictable long-term performance under enzymatic stress.

Peptoids, or poly-*N*-substituted glycines, are a class of peptide-mimetic, widely used to construct supramolecular nanomaterials.^17,18^ Synthesized via the solid-phase submonomer method using a broad range of monomers, peptoids allow for precise sequence definition and chemical tunability comparable to natural counterparts, peptides and proteins.^19,20^ These features enable the fine-tuning of self-assembly behavior and spatial control of functional moieties within the resulting peptoid assemblies.^21^ Moreover, peptoids exhibit exceptional proteolytic stability due to the placement of side chains on the nitrogen atom.^22^ These characteristics have enabled the development of protein-mimetic materials for diverse biomedical applications, including antimicrobial,^23,24^ cell cryopreservation,^25^ and molecular recognition agents.^26,27^ Despite these advantages, the development of supramolecular peptoid hydrogels presents ongoing challenges, with a limited number of hydrogel-forming peptoid sequences reported to date.^28–31^ This challenge reflects intrinsic limitations associated with peptoid self-assembly. Unlike peptides, peptoids lack backbone NH hydrogen bond donors, resulting in weaker intermolecular interactions and poor cohesive forces between peptoid strands.^32^ As a result, most existing peptoid hydrogels require external stimuli, such as temperature or pH changes, to induce network formation.^28,31^ These requirements complicate practical implementation by compromising the reproducibility of morphological and macroscopic properties of supramolecular gels.^33^

Herein, we present a minimal yet highly effective molecular engineering strategy that enables the spontaneous formation of supramolecular peptoid hydrogels as 3D-printable cell scaffolds and protease-stable cargo depots. Inspired by the enhanced self-assembly of peptides upon *N*-terminal alkylation, an octyl monomer was introduced at the *N*-terminus of a known peptoid hydrogelator.^34^ This simple molecular modification dramatically lowered the critical aggregation concentration by inducing hydrophobic collapse, enabling the spontaneous formation of highly ordered nanosheets without thermal or pH changes. These nanosheets further interconnected to form a supramolecular hydrogel network. The resulting hydrogels exhibited tunable viscoelasticity, shear-thinning, and self-healing properties, making them compatible with extrusion-based 3D printing. The printed constructs supported cell adhesion, spreading, and proliferation with high cell viability. Notably, the hydrogels retained their structural and mechanical integrity under proteolytic stress, enabling sustained cargo release with efficient cellular uptake. These findings highlight peptoids as a versatile platform for supramolecular hydrogels, integrating biocompatibility, bioactivity, and proteolytic stability. This work opens new avenues for the development of ECM-mimetic biomaterials tailored for long-term tissue engineering and drug delivery in protease-rich environments.

## 2 Results and Discussion

### 2.1 Rational Design of *N*-Terminal Octylated Peptoid Hydrogelators

Well-known peptoid hydrogelators are typically designed with a hydrophobic segment composed of four benzylamine monomers, (*Npm*)_4_, and a hydrophilic functional moiety at the *C*-terminus. A representative sequence, (*Npm*)_4_GRGD, where glycine (G) serves as a spacer and the RGD motif facilitates cell adhesion, has previously been shown to fold into nanosheets and form supramolecular gels in response to temperature change.^28^ However, (*Npm*)_4_GRGD exhibited immediate precipitation upon exposure to PBS buffer, and the resulting aggregates remained insoluble even after heating to 60 °C, suggesting that additional thermal input or modified conditions may be required for gelation. The necessity for gelation requiring thermal treatment inherently restricts its applicability in biological contexts due to the thermal instability of biomacromolecules.^35^ Furthermore, in the absence of temperature changes, (*Npm*)_4_GRGD failed to undergo gelation (Figure S1).

We hypothesized that the inability of (*Npm*)_4_GRGD to undergo hydrogelation stems from the lack of hydrogen bonding between peptoid backbones, which limits the driving forces required for supramolecular assembly and network formation. To address this limitation, we introduced an *N*-octylglycine monomer (*Noct*) at the *N*-terminus of (*Npm*)_4_GRGD to promote hydrophobic interactions, strengthen intermolecular cohesion, and presumably facilitate parallel chain alignment between peptoid strands (Figure 1a). Previous studies have demonstrated that *N*-terminal alkylation of peptides profoundly influences their aggregation behavior and secondary structure formation in aqueous environments across both synthetic and natural systems.^34,36^ Inspired by these findings, we postulated that introducing an alkyl tail at the *N*-terminus of the (*Npm*)_4_GRGD sequence would similarly enhance its self-assembly propensity. Specifically, the octyl group was expected to drive hydrophobic collapse between peptoid strands, while π–π stacking interactions between the pendant phenyl groups of *Npm* residues would contribute to structural ordering.^37,38^ Moreover, the resulting assemblies were anticipated to orient the hydrophilic GRGD motifs toward the interface, enabling cell adhesion. Guided by this design rationale, *Noct*(*Npm*)_4_GRGD was successfully obtained through peptoid synthesis and purification and confirmed by high-performance liquid chromatography and mass spectrometry (Figure S2).

**Figure 1:**
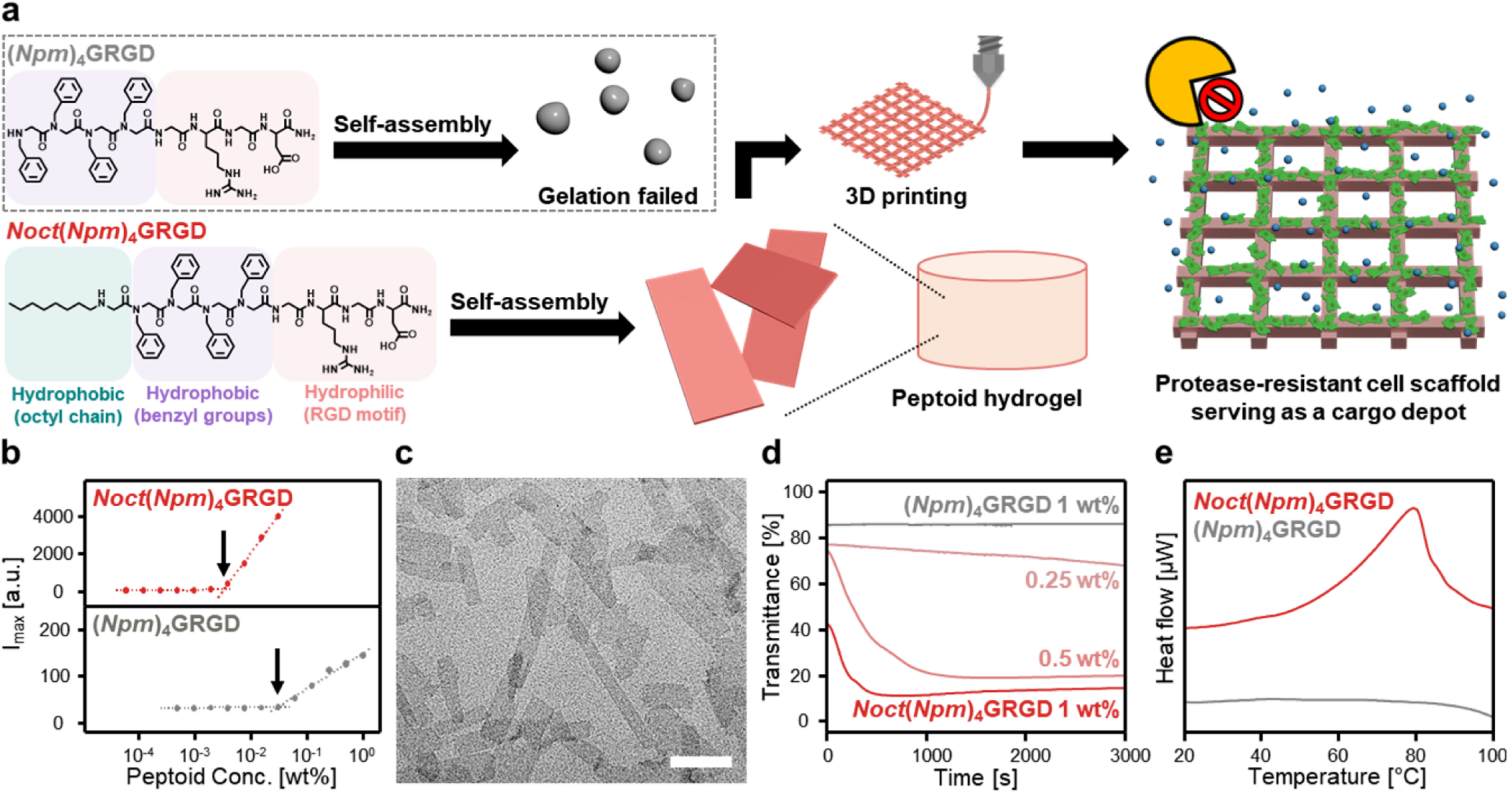
Enhanced peptoid self-assembly in the presence of an octyl monomer. (a) Chemical structures and schematic representations of the peptoid hydrogelators. *Noct*(*Npm*)_4_GRGD is an octyl-functionalized amphiphilic peptoid consisting of a hydrophobic alkyl tail (green), aromatic side chains (purple), and a hydrophilic RGD motif (red). This molecular design facilitates self-assembly into nanosheets in aqueous conditions, which subsequently form supramolecular hydrogels suitable for 3D printing. The resulting 3D-printed cell scaffolds support structural integrity and enable sustained cargo release under proteolytic stress. In contrast, the non-alkylated analogue (*Npm*)_4_GRGD fails to form hydrogels under the same aqueous conditions. (b) The degree of aggregation of *Noct*(*Npm*)_4_GRGD and (*Npm*)_4_GRGD as a function of peptoid concentration in water was determined by the ANS fluorescence assay. (c) TEM image of *Noct*(*Npm*)_4_GRGD at 1 wt % in water. The scale bar represents 500 nm. (d) Study of self-assembly kinetics using the transmittance change over time of 660 nm light through the solution. (e) Nano-DSC thermograms of *Noct*(*Npm*)_4_GRGD (red) and (*Npm*)_4_GRGD (gray) at 1 wt % in water.

### 2.2 Enhanced Peptoid Self-Assembly Through *N*-Terminal Octylation

To evaluate the effect of *Noct* on intermolecular cohesion of the peptoid strands, we determined the critical aggregation concentration (CAC) of *Noct*(*Npm*)_4_GRGD and (*Npm*)_4_GRGD using fluorescence spectroscopy (Figure 1b). 8-Anilino-1-naphthalene sulfonic acid (ANS) dye, a hydrophobicity-sensitive molecular probe, was employed to monitor the formation of hydrophobic domains.^39^ The CAC of *Noct*(*Npm*)_4_GRGD was determined to be approximately 3.3 × 10^–3^ wt %, nearly an order of magnitude lower than that of (*Npm*)_4_GRGD (3.0 × 10^–2^ wt %), indicating a markedly enhanced aggregation propensity upon *Noct* incorporation. This result was further corroborated by dynamic light scattering (DLS) analysis, which revealed a sharp, concentration-dependent increase in the derived count rate for *Noct*(*Npm*)_4_GRGD near its CAC, whereas (*Npm*)_4_GRGD exhibited consistently low count rates across all concentrations (Figure S3). Transmission electron microscopy (TEM) and cryogenic TEM (cryo-TEM) confirmed the formation of well-defined, sheet-like assemblies by *Noct*(*Npm*)_4_GRGD at 1 wt % in aqueous solution (Figure 1c and S4), in contrast to the spherical micellar aggregates observed for the unmodified sequence (Figure S5). Notably, aqueous solutions of 1 wt % *Noct*(*Npm*)_4_GRGD rapidly turned opaque within minutes, while those of (*Npm*)_4_GRGD remained transparent (Supplementary Movie 1). Turbidity measurements at 660 nm quantitatively supported this observation, showing a concentration-dependent increase in absorbance for the octylated peptoid, consistent with spontaneous self-assembly (Figure 1d). Solution-phase differential scanning calorimetry (Nano-DSC) provided further evidence for ordered assembly. *Noct*(*Npm*)_4_GRGD exhibited a distinct endothermic transition at 79.5 °C with an associated enthalpy (ΔH) of 46.36 kJ mol^–1^ and entropy (ΔS) of 0.13 kJ mol^–1^ K^–1^, whereas (*Npm*)_4_GRGD displayed no detectable thermal transition (Figure 1e). This observation suggests that the *Noct*(*Npm*)_4_GRGD spontaneously assembles into highly ordered nanostructures.

### 2.3 Supramolecular Organization of *N*-Terminal Octylated Peptoids

To gain deeper insight into the molecular packing and structural organization of *Noct*(*Npm*)_4_GRGD assemblies, small- and wide-angle X-ray scattering (SAXS and WAXS) analyses were conducted (Figure 2a and S6). The scattering profile of *Noct*(*Npm*)_4_GRGD nanosheets revealed two sharp diffraction peaks at 14.6 Å and 4.7 Å, whereas (*Npm*)_4_GRGD aggregates displayed no discernible peaks across the entire scattering range. These results clearly indicated that *Noct*(*Npm*)_4_GRGD strands assembled into a highly ordered supramolecular structure, in stark contrast to the disordered aggregates of their unmodified counterparts. The observed 4.7 Å peak was assigned to the lateral intermolecular distance between peptoid backbones (*a*-spacing), while the 14.6 Å peak was attributed to the inter-row distance (*c*-spacing), likely arising from π–π interactions between aromatic *Npm* side chains.^30^ These distances are commensurate with the known lattice parameters of previously reported crystalline peptoid assemblies composed of extended chains in the sigma strand conformation.^40^ Atomic force microscopy (AFM) imaging further confirmed the formation of nanosheets with a uniform, planar morphology and an average height of 5.30 ± 0.17 nm (Figure S7). This nanosheet thickness correlated well with the broad SAXS peak at 41.9 Å.

**Figure 2:**
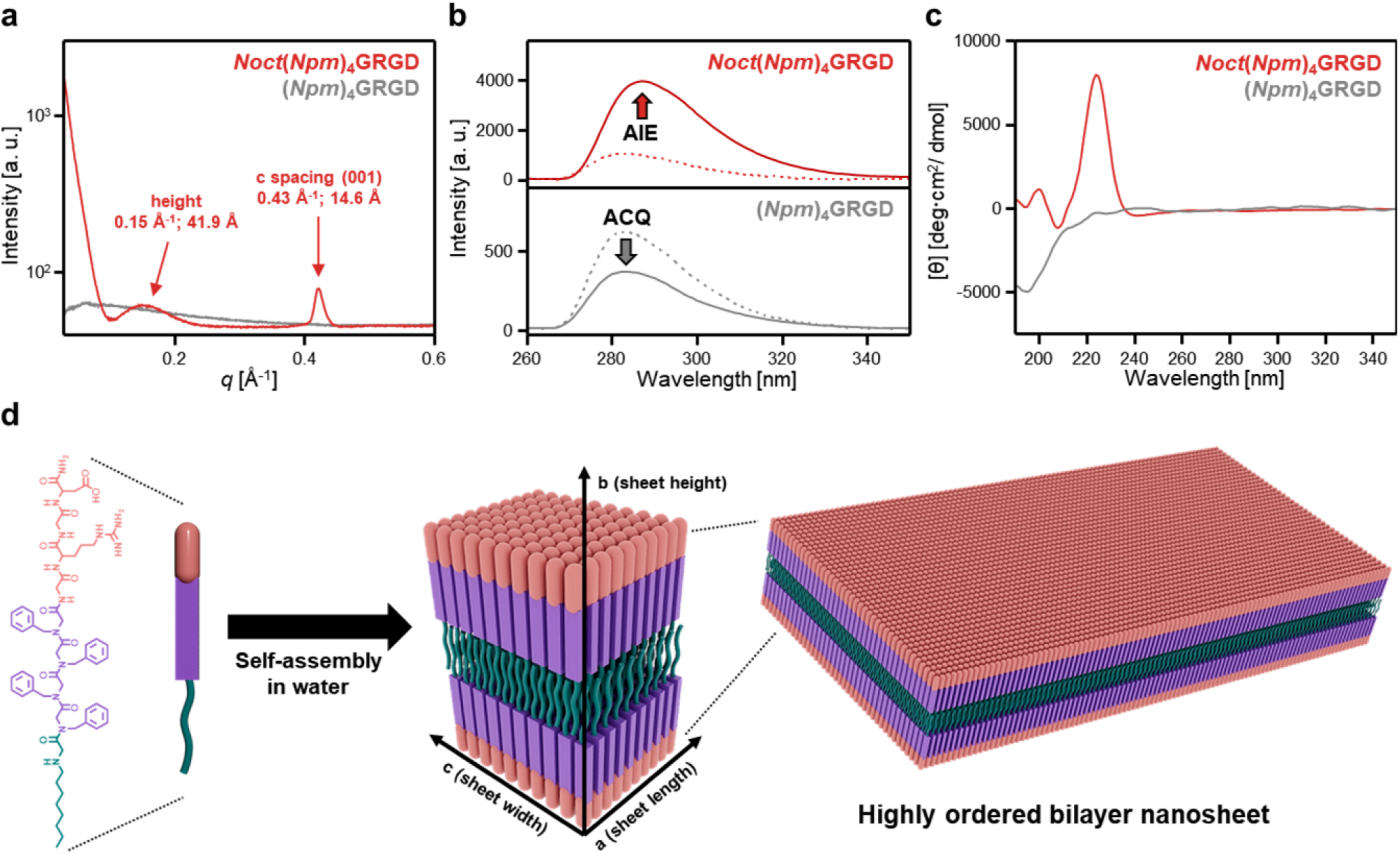
Structural characterization of self-assembled *Noct*(*Npm*)_4_GRGD nanostructures. In typical conditions, 1 wt % of peptoid strands were dissolved in the desired solvents. (a) Solution-SAXS profiles of *Noct*(*Npm*)_4_GRGD (red) and (*Npm*)_4_GRGD (gray). (b) PL spectra of *Noct*(*Npm*)_4_GRGD (red) and (*Npm*)_4_GRGD (gray) in water (solid line) and a 50:50 (v/v) ACN/water mixture (dashed line) excited at 254 nm. (c) CD spectra of *Noct*(*Npm*)_4_GRGD (red) and (*Npm*)_4_GRGD (gray). (d) Schematic model of the proposed molecular assembly for *Noct*(*Npm*)_4_GRGD. The nanostructure is stabilized by lateral alignment of aromatic side chains along the *a*-direction (sheet length), layered packing along the *c*-direction (sheet width), and vertical stacking of interdigitated alkyl tails along the *b*-direction (sheet height), resulting in a crystalline bilayer architecture with nanoscale precision.

Photoluminescence (PL) spectroscopy provided additional evidence for the ordered molecular packing within *Noct*(*Npm*)_4_GRGD nanosheets (Figure 2b). In aqueous solution, *Noct*(*Npm*)_4_GRGD exhibited a significant increase in PL intensity compared to its unassembled state in a 50:50 (v/v) acetonitrile (ACN)/water mixture. This enhancement was attributed to the aggregation-induced emission (AIE) effect, where restricted molecular motion and suppressed nonradiative decay result from π–π interactions between benzyl side chains.^41,42^ In contrast, (*Npm*)_4_GRGD displayed reduced PL intensity in water, consistent with aggregation-caused quenching (ACQ), a phenomenon associated with disordered aggregates characterized by dynamic molecular motion and nonradiative relaxation.^41^ The *N*-terminal octyl group promoted hydrophobic collapse and the formation of densely packed nanosheets. This ordered packing restricted intramolecular rotations of the aromatic *Npm* residues, suppressing nonradiative relaxation and thereby enhancing radiative decay. As a result, *Noct*(*Npm*)_4_GRGD exhibited AIE accompanied by a red-shift and spectral broadening. In contrast, (*Npm*)_4_GRGD assembled into loosely packed, disordered aggregates that lacked sufficient packing constraints. The freedom of intramolecular motion favored nonradiative decay, consistent with ACQ and the absence of spectral broadening. These distinct photophysical behaviors were further supported by UV–Vis absorption spectroscopy (Figure S8). *Noct*(*Npm*)_4_GRGD exhibited broader and red-shifted π–π* and n−π* transition peaks near 225 nm in water compared to the ACN/water mixture, indicating densely packed benzene rings within highly ordered supramolecular nanostructures.^43^ In contrast, (*Npm*)_4_GRGD showed minimal spectral changes, suggesting weak or disordered aggregation. Notably, *Noct*(*Npm*)_4_GRGD exhibited a concentration-dependent red-shift in its PL spectrum, moving from 281 to 287 nm in aqueous solution (Figure S9). This bathochromic shift is characteristic of J-aggregate formation, where aromatic moieties align in a head-to-tail configuration.^44^ This shift was absent in (*Npm*)_4_GRGD, further highlighting its disordered aggregation behavior. Circular dichroism (CD) spectroscopy revealed the emergence of supramolecular chirality associated with ordered assemblies. While (*Npm*)_4_GRGD exhibited a weak negative peak at 196 nm, indicative of a largely disordered structure, *Noct*(*Npm*)_4_GRGD displayed a Cotton effect, with a strong positive peak at 224 nm and a weak negative peak at 241 nm (Figure 2c). These features likely arise from exciton coupling in n−π* transition, stemming from backbone ordering and conformational rigidity associated with J-aggregation-like domains.^45^ The observed chiroptical response may also be influenced by the stereochemistry of the GRGD sequence^46^ and the amphiphilic environment introduced by the *N*-terminal octyl group.^47,48^ Based on these combined structural analyses, we propose that *Noct*(*Npm*)_4_GRGD spontaneously assembles into a highly ordered bilayer nanosheet architecture (Figure 2d).

In this model, peptoid strands align laterally with an intermolecular distance of 4.7 Å (*a*-direction), while vertical stacking occurs along the *c*-direction with an interlayer spacing of 14.6 Å, consistent with directional π–π interactions between pendant aromatic moieties adopting a J-aggregate-like configuration. The fully extended molecular length of *Noct*(*Npm*)_4_GRGD was estimated to be approximately 3.8 nm (Figure S10). Given this molecular length and the average nanosheet thickness of approximately 5 nm observed by AFM, we inferred an interdigitated bilayer configuration in which hydrophobic octyl chains from opposing layers interpenetrate. This packing arrangement minimizes energetically unfavorable voids and reduces electrostatic repulsion between GRGD-bearing surfaces, thereby stabilizing the lamellar assembly through a combination of hydrophobic collapse and π–π interactions.^49^

### 2.4 3D-Printable Peptoid Hydrogels

Given its ability to spontaneously self-assemble into highly ordered nanosheets, we next investigated the hydrogelation behavior of *Noct*(*Npm*)_4_GRGD. Inverted vial tests across varying concentrations identified a critical gelation concentration (CGC) of 1.5 wt % (Figure 3a). Consistent with its rapid self-assembly, oscillatory time-sweep rheology showed that gelation occurred within a few minutes after dissolution, with storage modulus (G′) rapidly surpassing loss modulus (G″) and continuing to increase as the network matured (Figure S11). Scanning electron microscopy (SEM) of the resulting hydrogels revealed a highly porous network architecture composed of densely stacked nanosheets that were not merely layered, but also interconnected via ribbon-like domains. This morphology was enabled by the fluidity of the alkyl segment and the intrinsic flexibility of the peptoid backbone (Figure 3b).^38^ This hierarchical structure is expected to enhance both the structural integrity and mechanical stability of the hydrogel.^50^

**Figure 3:**
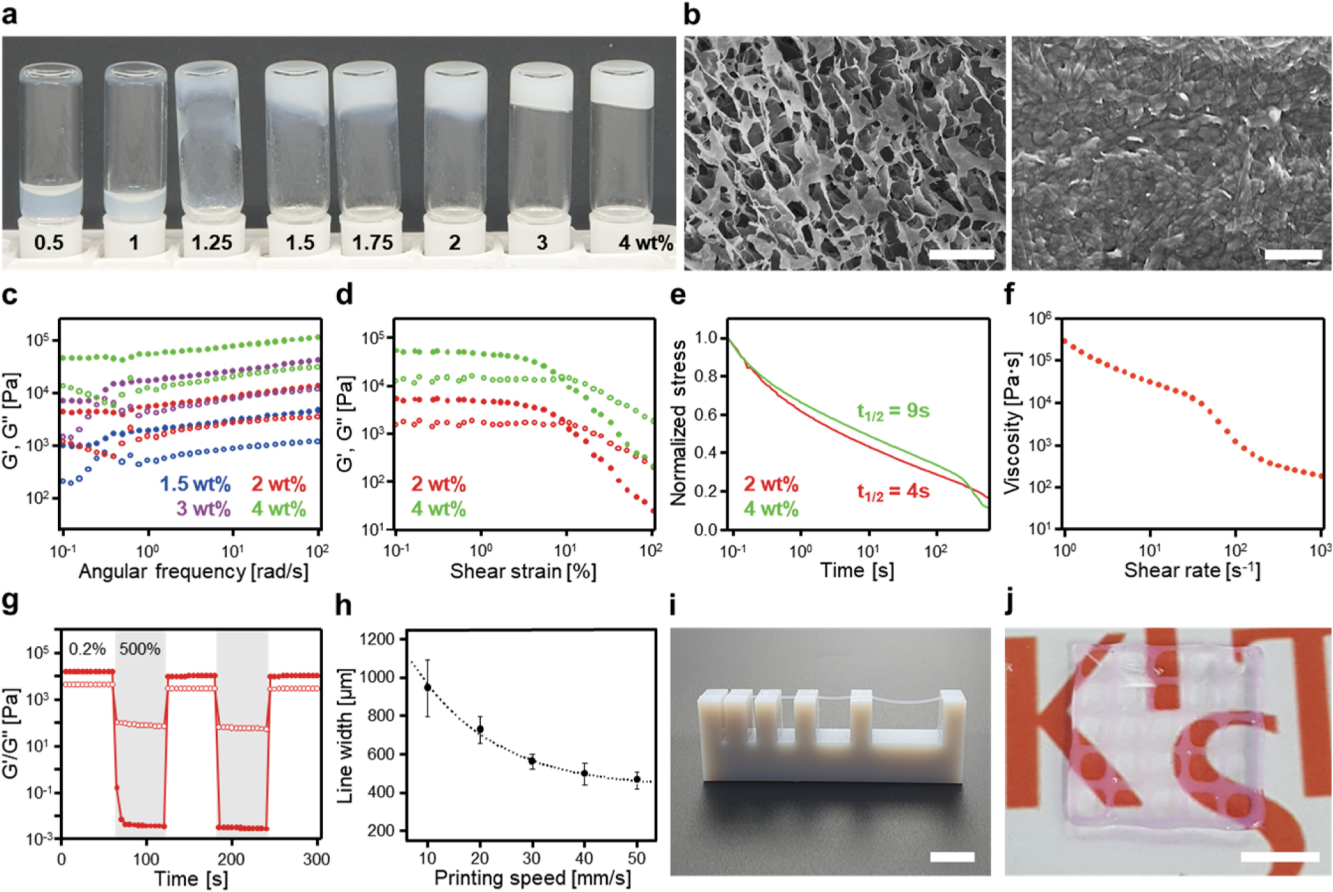
Structural and rheological characterization of *Noct*(*Npm*)_4_GRGD hydrogels and their 3D printability. In typical experiments for printability, peptoid hydrogels were prepared at the concentration of 2 wt % *Noct*(*Npm*)_4_GRGD. (a) Inverted vial test demonstrating concentration-dependent hydrogel formation from 0.5 to 4 wt % of *Noct*(*Npm*)_4_GRGD. (b) SEM images of supramolecular peptoid hydrogels formed by 2 wt % *Noct*(*Npm*)_4_GRGD in water. The scale bars represent 10 μm (left) and 500 nm (right). (c) Frequency sweep test at 0.1% strain for *Noct*(*Npm*)_4_GRGD hydrogels at different concentrations. (d) Amplitude sweep test at 1 rad/s for *Noct*(*Npm*)_4_GRGD hydrogels. (e) Normalized stress relaxation curves of *Noct*(*Npm*)_4_GRGD hydrogels obtained under the applied 10% strain. (f) Shear thinning and (g) self-healing behavior of *Noct*(*Npm*)_4_GRGD hydrogels. G′ and G″ were monitored under alternating shear strains of 0.2% and 500% at 1 rad/s. All rheological plots display G′ as filled circles and G″ as open circles. (h) Printing speed calibration curve for *Noct*(*Npm*)_4_GRGD hydrogels. Line width was measured 12 times (*n* = 12) and averaged. (i) Overhang test for *Noct*(*Npm*)_4_GRGD hydrogels. The scale bar represents 10 mm. (j) Optical image of a multilayer 3D-printed mesh structure after PBS buffer incubation. The scale bar represents 5 mm.

To assess how the nanosheet-based architecture influences macroscopic properties, we performed rheological characterizations. Frequency sweep measurements showed a concentration-dependent increase in both G′ and G″, with G′ increasing from 2 kPa at 1.5 wt % to 53 kPa at 4 wt % (Figure 3c), confirming stiffness tunability, an essential feature for tailoring hydrogel scaffolds to match the mechanical requirements of various soft tissues.^5^ Amplitude sweep tests showed a linear viscoelastic region extending up to approximately 3% strain, with a flow point below 10% strain (Figure 3d). Stress relaxation experiments under 10% constant strain showed rapid dissipation of over half the initial stress (*t*_1/2_) within 10 s (Figure 3e). The low-strain yielding behavior and rapid stress relaxation are hallmark characteristics of ECMs.^51^ In addition to these mechanical characteristics, the hydrogels maintained stable properties under physiologically relevant environmental conditions. Isothermal frequency-sweep measurements performed within the gel-state temperature range (25–55 °C) showed negligible changes in G′ and G″, indicating temperature-independent viscoelastic behavior (Figure S12). Similarly, hydrogels formed reliably across pH 5–8 (Figure S13), indicating that the nanosheet-based network remains intact throughout this physiologically relevant pH range.

We further characterized rheological properties relevant to extrusion-based 3D printing, a prerequisite for fabricating spatially defined, bioactive scaffolds.^52,53^ Shear rate-dependent viscosity measurements confirmed shear-thinning behavior, enabling smooth flow under applied stress (Figure 3f). Interval thixotropy tests demonstrated self-healing capability, with full recovery of mechanical stiffness following repeated cycles of yielding and recovery (Figure 3g). Based on these shear-thinning and self-healing properties, we comprehensively evaluated the 3D printability of *Noct*(*Npm*)_4_GRGD hydrogels through a series of extrusion-based printing experiments. Printed line widths were tunable by adjusting the printing speed, with calibration curves closely fitting an exponential decay model (R^2^ = 0.98) (Figure 3h). Filaments extruded through a 21G nozzle exhibited a height and width of 500 μm, consistent with the nozzle’s inner diameter. Overhang tests demonstrated structural retention at spans of 1, 2, 4, 8, and 16 mm, confirming the self-supporting ability of the peptoid hydrogels (Figure 3i). Under optimized conditions, we successfully printed a planar mesh with uniform filaments and pore sizes of 1.5–1.7 mm (Figure 3j), which retained its shape after incubation in PBS buffer. Taken together, these results position *Noct*(*Npm*)_4_GRGD hydrogels as a versatile platform for fabricating structurally stable, mechanically tunable, and 3D-printable scaffolds.

### 2.5 Biocompatibility and Cell-Supporting Properties of Peptoid Hydrogels

To assess the suitability of *Noct*(*Npm*)_4_GRGD hydrogels as cell scaffolds, we evaluated their ability to support NIH-3T3 fibroblast viability, adhesion, and proliferation. Fluorescence microscopy revealed dynamic cellular responses over time (Figure 4a). As early as 6 h post-seeding, fibroblasts exhibited well-spread morphologies with lamellipodia, indicating successful adhesion to the hydrogel matrix. As culture progressed, cells extended filopodia-like protrusions and increasingly interacted with neighboring cells. By day 4, the emergence of localized cell clusters suggested that the hydrogel not only maintained cell viability but also supported early stage cell–cell interactions and tissue-like organization.^54,55^ A live/dead cytotoxicity assay confirmed high cytocompatibility, with cell viability exceeding 95% after 4 days in culture (Figure 4b). Quantitative analysis further confirmed a steady increase in cell density over time, reflecting continued cell proliferation on the peptoid hydrogel (Figure 4c). These cellular behaviors were consistently observed on 3D-printed mesh constructs, highlighting their potential as customizable cell scaffolds (Figure 4d). By day 1, fibroblasts were well-attached and showed initial spreading across the printed hydrogel. By day 4, prominent cell clusters and enhanced cytoskeletal activity were evident, as evidenced by extensive lamellipodia and filopodia extensions.^56^ These features were indicative of active migration and collective behavior, suggesting that the printed scaffold supported spatial organization and early tissue-like assembly. Although the hydrogels supported stable adhesion and proliferation, the extent of cell spreading remained moderate. This behavior is consistent with previous reports showing that excessively high ligand densities can hinder integrin organization, suppress protrusion formation, and ultimately limit spreading.^57^ Hu and coworkers further demonstrate that ligand presentation can be tuned through coassembly, suggesting a potential strategy to enhance spreading in our system.

**Figure 4:**
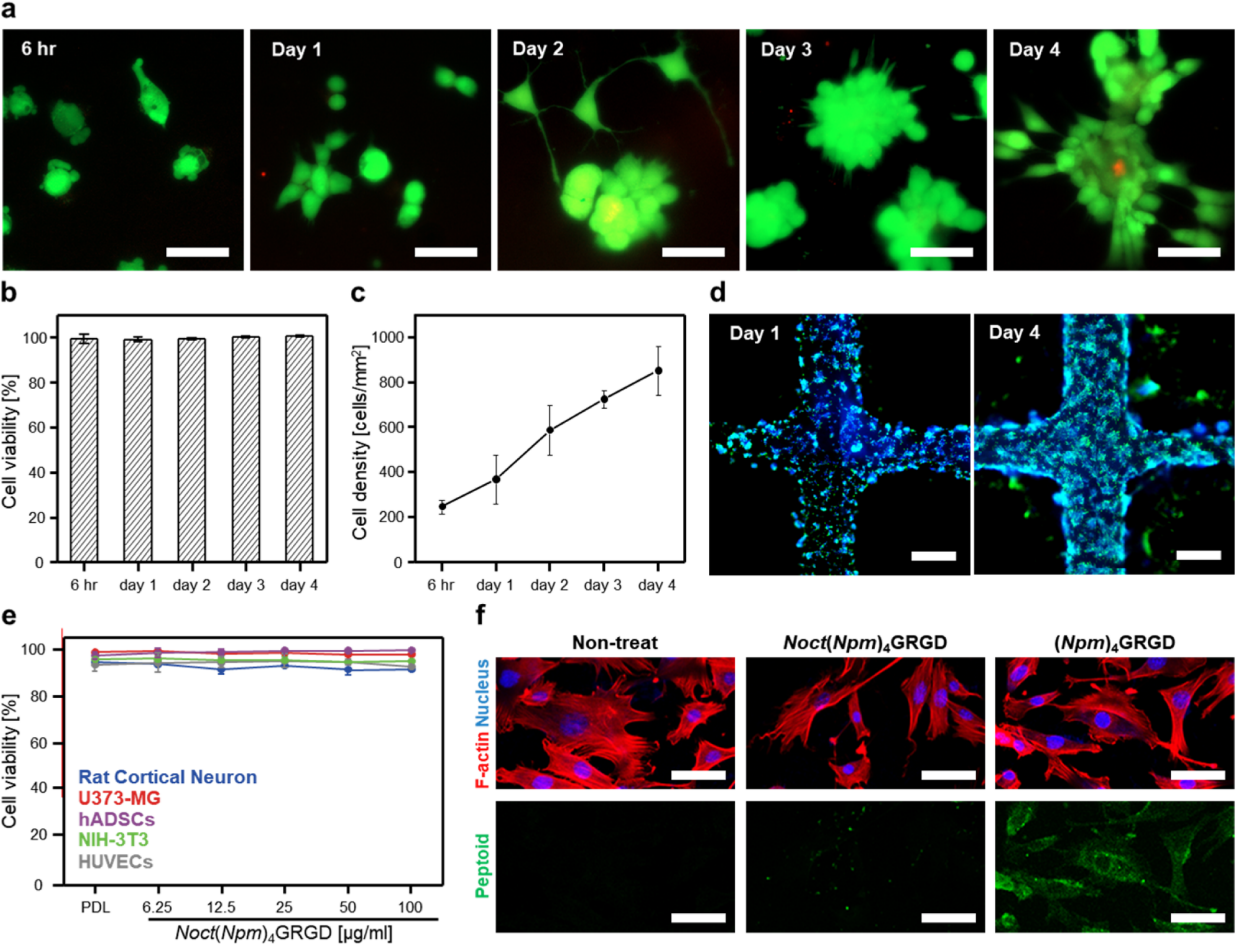
Biocompatibility of *Noct*(*Npm*)_4_GRGD hydrogels as cell scaffolds. (a) Fluorescent microscopic images of NIH-3T3 fibroblasts cultured on *Noct*(*Npm*)_4_GRGD hydrogels over time. Cells stained with Calcein AM (live, green) and EthD-1 (dead, red). The scale bars represent 50 μm. (b) Quantification of cell viability and (c) proliferation based on time-dependent cell counts using fluorescent microscopic images. All values were measured four times (*n* = 4) and averaged. Cell counts were obtained from five randomly selected fields per sample, each corresponding to ∼6.30 mm^2^ of imaging area. (d) Representative fluorescence microscopic images of NIH-3T3 fibroblasts cultured on 3D-printed mesh structures of 2 wt % *Noct*(*Npm*)_4_GRGD hydrogel at day 1 (left) and day 4 (right). F-actin and nucleus were stained by phalloidin rhodamine (green) and DAPI (blue), respectively. The scale bars represent 500 μm. (e) Cytotoxicity assay for *Noct*(*Npm*)_4_GRGD strands across various cell lines, including rat cortical neurons (blue), human astrocytoma (U373-MG, red), human adipose-derived stem cells (hADSCs, purple), mouse fibroblast (NIH-3T3, green) human umbilical vein endothelial cells (HUVECs, gray) (*n* = 4). Poly-d-lysine (PDL) was used as a control. All values were measured four times and averaged. (f) Representative fluorescence microscopic images showing cellular internalization of (*Npm*)_4_GRGD aggregates and *Noct*(*Npm*)_4_GRGD nanosheets. Peptoids were labeled with FAM (green) at the C-terminus via an additional lysine residue. F-actin and nucleus were stained by phalloidin rhodamine (red) and DAPI (blue), respectively. The scale bars represent 100 μm.

Beyond supporting cell viability, adhesion, and proliferation, we systematically evaluated the molecular safety and stability of *Noct*(*Npm*)_4_GRGD to determine its suitability as a cell scaffold. Cytotoxicity screening of *Noct*(*Npm*)_4_GRGD across a panel of cell lines, including neural (rat cortical neurons), glial (U373-MG), mesenchymal stem (hADSCs), fibroblast (NIH-3T3), and vascular endothelial (HUVECs) cells, revealed negligible cytotoxic effects even at 86.2 μM (100 μg/mL), a concentration that lies within the upper micromolar range commonly used for peptoid biocompatibility studies^58,59^ (Figure 4e). Given that supramolecular hydrogels are stabilized by noncovalent interactions, we sought to assess the potential for disassembly and subsequent internalization of *Noct*(*Npm*)_4_GRGD nanosheets, as such events could affect cell behavior and compromise extracellular stability. Fluorescence imaging revealed that while (*Npm*)_4_GRGD aggregates accumulated intracellularly, *Noct*(*Npm*)_4_GRGD nanosheets remained predominantly extracellular (Figure 4f). This reduced cellular uptake is consistent with previous studies showing that high-aspect-ratio nanostructures exhibit lower internalization rates due to unfavorable membrane wrapping energetics, which disfavor endocytosis and promote extracellular stability.^60–63^ The planar morphology of our nanosheets introduced an energy barrier for endocytosis compared to the spherical aggregates formed by (*Npm*)_4_GRGD. Such minimized cell internalization may be advantageous for applications requiring sustained extracellular bioactivity.

### 2.6 Proteolytically Robust Peptoid Hydrogels as Sustained Cargo Depots

Building on their function as biocompatible scaffolds with prolonged extracellular residence, we investigated the structural and mechanical integrity of *Noct*(*Npm*)_4_GRGD hydrogels under enzymatic stress. Proteolytic resistance is particularly advantageous in protease-rich environments such as chronic wounds, inflammatory tissues, and tumor microenvironments, where scaffold stability is critical for long-term tissue regeneration and controlled drug delivery.^64,65^ Upon exposure to proteinase K, *Noct*(*Npm*)_4_GRGD strands remained structurally intact after 24 h (Figure S14). In contrast, Fmoc-diphenylalanine (Fmoc-FF), a well-known peptide hydrogelator, degraded completely within 1 h, producing byproducts known to trigger necrosis and inflammatory responses.^14^ To assess hydrogel-level stability in proteolytic conditions, *Noct*(*Npm*)_4_GRGD and Fmoc-FF hydrogels were incubated with proteinase K and monitored over time (Figure 5a). While Fmoc-FF hydrogels rapidly disintegrated, *Noct*(*Npm*)_4_GRGD hydrogels retained their macroscopic shape. Quantitative mass retention analysis revealed substantial degradation of Fmoc-FF hydrogels, whereas *Noct*(*Npm*)_4_GRGD hydrogels maintained nearly full mass after 14 days of continuous enzymatic exposure (Figure 5b). In addition to structural stability against proteinase K, *Noct*(*Npm*)_4_GRGD hydrogels also retained their mechanical properties under proteolytic stress. Frequency sweep tests demonstrated stable storage modulus (G′) of *Noct*(*Npm*)_4_GRGD hydrogels after prolonged proteinase K exposure, in contrast to the progressive decline observed in Fmoc-FF hydrogels, indicating network degradation and mechanical softening (Figure 5c). Following confirmation of proteolytic stability of *Noct*(*Npm*)_4_GRGD hydrogel matrix, we evaluted the cargo release behavior of *Noct*(*Npm*)_4_GRGD hydrogels under proteolytic stress. Release studies using 4 kDa FITC-dextran as a model cargo revealed that both *Noct*(*Npm*)_4_GRGD and Fmoc-FF hydrogels followed sustained release kinetics (Figure 5d), with strong linearity to the Higuchi diffusion model^66^ (R^2^ > 0.97) under all conditions (Figure S15). However, in the presence of proteinase K, the two hydrogels exhibited markedly different release profiles. *Noct*(*Npm*)_4_GRGD hydrogels maintained consistent release kinetics, with minimal changes in T50% and T70%, consistent with passive diffusion from an intact matrix (Figure 5e). In contrast, Fmoc-FF hydrogels showed accelerated cargo release rate in T50% and T70% due to network degradation. The ability of *Noct*(*Npm*)_4_GRGD hydrogels to preserve cargo release behavior under proteolytic stress suggested their potential for long-term cargo delivery applications.

**Figure 5:**
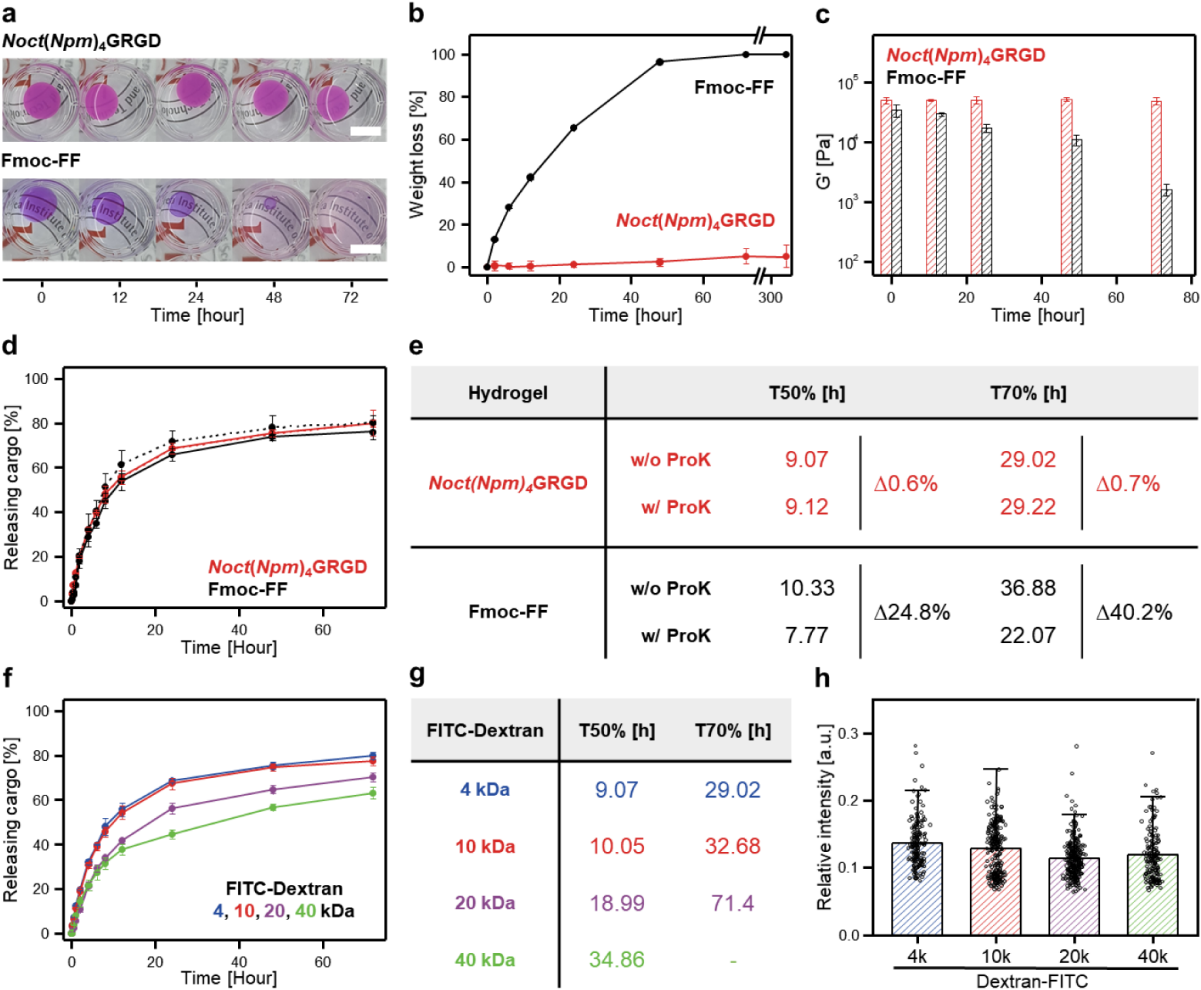
Proteolytic robustness and sustained cargo delivery of *Noct*(*Npm*)_4_GRGD hydrogels. (a) Macroscopic observation and (b) quantification of proteolytic degradation for *Noct*(*Npm*)_4_GRGD (red) and Fmoc-FF (black) hydrogels in Tris-HCl buffer containing 0.2 mg/mL of proteinase K over time (*n* = 3). Both hydrogels were stained by Nile Red. The scale bars represent 1 cm. (c) Time-course storage modulus (G′) measurements for *Noct*(*Npm*)_4_GRGD (red) and Fmoc-FF hydrogels (black) in the presence of proteinase K (*n* = 3). (d) Cargo release kinetics for 4 kDa FITC-dextran from *Noct*(*Npm*)_4_GRGD (red) and Fmoc-FF hydrogels (black) in the presence (dashed line) and absence (solid line) of proteinase K (*n* = 3), and (e) the corresponding T50% and T70% values with percentage changes, calculated by linear interpolation. (f) Cumulative release profiles of FITC-dextran cargos with molecular weights of 4 kDa (blue), 10 kDa (red), 20 kDa (purple), and 40 kDa (green) from *Noct*(*Npm*)_4_GRGD hydrogels (*n* = 3), and (g) the corresponding T50% and T70% values, calculated by linear interpolation. (h) Quantification of intracellular fluorescence intensities of NIH-3T3 fibroblast cells cultured for 24 h on *Noct*(*Npm*)_4_GRGD hydrogels loaded with FITC-dextran cargos of 4 kDa (blue), 10 kDa (red), 20 kDa (purple), and 40 kDa (green) (*n* = 4). Fluorescence intensities were quantified from five random fields per sample.

To further probe hydrogel network permeability, we tested dextran cargos ranging from 4 to 40 kDa, representing the size spectrum of common biomacromolecules such as cytokines and growth factors.^67,68^ The release profiles revealed size-dependent diffusion, with larger cargos releasing more slowly (Figure 5f). Notably, all cargo types exhibited sustained release, with T50% values exceeding 8 h (Figure 5g). To assess cellular uptake, fibroblasts were cultured on FITC-dextran-loaded *Noct*(*Npm*)_4_GRGD hydrogels. After 24 h, intracellular green granules were observed for all dextran sizes (Figure S16), confirming uptake of released cargos via endocytic uptake pathways.^69^ Quantitative analysis further demonstrated efficient cellular internalization regardless of cargo size under steady-state conditions (Figure 5h). These results collectively demonstrate that *Noct*(*Npm*)_4_GRGD hydrogels act as proteolytically stable cargo depots suitable for sustained delivery in protease-rich environments.

## 3 Conclusions

In this study, we report an *N*-terminal octyl peptoid self-assembly strategy for constructing supramolecular hydrogels that function as both 3D-printable scaffolds and proteolytically stable cargo depots. Incorporation of an *N*-terminal octyl monomer into the peptoid sequence *Noct*(*Npm*)_4_GRGD promoted spontaneous folding into highly ordered nanosheets via synergistic hydrophobic collapse of the octyl tails and π–π stacking of aromatic side chains. The flexibility of the peptoid backbone, combined with partial mobility of the octyl domain, further facilitated the formation of interconnecting ribbon-like structures that enabled robust hydrogelation. Above the CGC of 1.5 wt %, the interconnected hydrogel networks exhibited tunable viscoelasticity, shear-thinning, and self-healing behavior, making them suitable for extrusion-based 3D printing.

The resulting hydrogels demonstrated exceptional biocompatibility, supporting cell adhesion, spreading, and proliferation with over 95% cell viability across extended culture periods. Furthermore, their inherent enzymatic resistance enabled sustained cargo diffusion and effective cellular uptake even under protease-rich conditions that rapidly degrade conventional peptide-based hydrogels. This combination of proteolytic stability, structural fidelity, and controlled release positions supramolecular peptoid hydrogels as a promising alternative to existing biohydrogels for long-term applications. As versatile and robust ECM-mimetic biomaterials, they offer new opportunities for long-term tissue regeneration, precision drug delivery, and biofabrication in pathologically challenging environments.

## 4 Methods

### 4.1 Materials

Rink amide MBHA resin (100–200 mesh, 0.65 mmol/g), Fmoc-Gly-OH (98%), Fmoc-Lys­(Mtt)-OH (98%), Fmoc-Arg­(Pbf)-OH (98%), Fmoc-Asp­(OtBu)-OH (98%), triisopropylsilane (TIPS, 98%), HBTU (98%), Diisopropylethylamine (DIPEA, 99%) trifluoroacetic acid (TFA, 99%), 5(6)-carboxyfluorescein (FAM, 95%), fluorescein isothiocyanate–dextran (FITC-Dextran, average molecular weight 4, 10, 20, and 40 kDa), Nile Red (97%), and Proteinase K (from *Tritirachium album*) were purchased from Sigma-Aldrich (USA). n-Octylamine (98%), benzylamine (99%), and *N,N*′-Diisopropylcarbodiimide (DIC, 98%) were purchased from TCI Chemicals (Japan). Bromoacetic acid (98%), PBS buffer (10×, pH 7.4), Tris-HCl buffer (1 M, pH 8.0), Phalloidin rhodamine (R415) and the LIVE/DEAD viability/cytotoxicity kit were purchased from Thermo Fisher Scientific (USA). Fmoc-Phe-Phe-OH (Fmoc-FF, 99%) was purchased from Bachem (Switzerland). All reagents were used as received without further purification unless otherwise specified. Milli-Q water was used for all HPLC purification and sample preparation steps.

### 4.2 Peptoid Synthesis and Purification

Peptoid sequences were synthesized using a modified solid-phase submonomer method as previously described.^19^ Briefly, peptide sequences were constructed on Rink amide MBHA resin (0.1 mmol) via standard Fmoc-based solid-phase peptide synthesis (SPPS), using repeated cycles of Fmoc deprotection with 20% piperidine in DMF for 30 min, followed by amino acid coupling using Fmoc-protected amino acids (0.12 M), HBTU (0.11 M), and DIPEA (0.24 M) in DMF for 3 h. Peptoid monomers were then incorporated by submonomer synthesis: bromoacetylation with bromoacetic acid (0.8 M) and DIC (0.8 M) in DMF for 20 minutes, followed by nucleophilic displacement with the desired amine monomer (1 M) in DMF for 30 minutes. For deprotection and cleavage, the resin was treated with a cleavage cocktail (TFA:water:TIPS = 97:2:1) for 3 h, and the crude product was concentrated by N_2_ blowing. Peptoid hydrogelators were purified using reversed-phase HPLC (water-ACN with 0.1% TFA) using a C18 column (Sunfire; 5 μm, 19 × 150 mm) and characterized by UPLC and MALDI-TOF mass spectrometry (Figure S2). The FAM-labeled peptoid hydrogelators were synthesized by incorporating a lysine residue at the *C*-terminal GRGD motif, followed by conjugation of 5(6)-carboxyfluorescein to its side chain. All labeled compounds were characterized by HPLC and MALDI-TOF (Figure S17). All peptoids used in this study had a purity greater than 95%.

### 4.3 Hydrogel Preparation

*Noct*(*Npm*)_4_GRGD was dissolved in Milli-Q water, and hydrogelation occurred spontaneously within minutes at room temperature. For Fmoc-FF hydrogels, a 100 mg/mL stock solution of Fmoc-FF in DMSO was slowly added dropwise into Milli-Q water under gentle vortexing, resulting in immediate gelation. After gelation, residual DMSO was removed by washing the hydrogels in Milli-Q water for 1 h with three water exchanges.

### 4.4 Characterization

UV–Vis, PL, and CD spectra were measured using V-670, FP-8500, and J-1100 instruments, respectively (JASCO, Japan). CAC was determined by adding 2 μL of 2 wt % ANS solution in DMSO to 2 mL of peptoid aqueous solution (0.1 vol % DMSO), followed by 5 s vortexing and PL measurement at 356 nm excitation. DLS measurements (Zetasizer Nano ZS; Malvern Instruments, UK) were performed to evaluate the derived count rate of the peptoid assemblies (*n* = 3). Nano-DSC was conducted on a CSC 6100 (TA Instruments, USA) from 20 to 100 °C at a heating rate of 1 °C/min. SAXS and WAXS data were collected at Beamline 4C of the Pohang Accelerator Laboratory (PAL, Republic of Korea),^70^ using a synchrotron X-ray source (16.9 keV, beam size 100 μm × 30 μm) and a Rayonix SX165 CCD detector. Data were collected at sample-to-detector distances of 1 m and 20 cm with 30 s exposure time and processed using FIT2D (ESRF). Samples were sealed in quartz capillaries (Hilgenberg, Germany) and analyzed at room temperature. TEM (JEM-2100Plus; JEOL, Japan) was performed at 200 kV on uranyl acetate-stained (0.4 wt %) samples drop-cast onto carbon-coated copper grids. Cryo-TEM (Tecnai F20 G2; FEI, USA) was performed at 120 kV on vitrified samples prepared using a PIPS system (Gatan, USA) and imaged under cryogenic conditions. FE-SEM (Sigma 300, Zeiss) was conducted on lyophilized hydrogel coated with ∼4 nm Pt using a sputter coater (Cressington Scientific Instruments, UK). AFM (XE-7; Park Systems, Republic of Korea) was performed in noncontact mode on peptoid hydrogelator film drop-cast and dried on mica, and height profiles were obtained from independent line scans (*n* = 10). The temperature stability of hydrogels (preformed for 24 h at 25 °C) was evaluated using the inverted-vial method. Samples were heated stepwise, incubated for 10 min at each temperature to reach equilibrium, and then assessed by inversion. When a sol–gel transition was detected, the samples were cooled to 25 °C, equilibrated for 10 min, and retested. For pH-dependent hydrogelation experiment, hydrogels were prepared in aqueous solutions adjusted to pH 5–8 using dilute HCl or NH_4_OH. After mixing, samples were allowed to equilibrate for 1 h at room temperature, and gelation was evaluated using the inverted-vial method. MALDI-TOF was performed using an Ultraflextreme system (Bruker, Germany). UPLC was carried out on a Waters Acquity system with a BEH C18 column (2.1 × 100 mm, 1.7 μm, 300 Å, Waters Corporation, USA). Analytical HPLC was conducted using a Water system (2489 UV detector, 1525 pump, 2707 autosampler) with a SunFire C18 column (4.6 × 250 mm, 5 μm, Waters Corporation, USA).

### 4.5 Rheology

Rheological measurements were performed using an MCR 302e rheometer (Anton Paar, Austria) equipped with a 25 mm parallel plate geometry. Approximately 200 μL of hydrogel was loaded onto the stage, and the upper plate was adjusted to a fixed 0.5 mm gap. Time-sweep rheology was performed using freshly prepared peptoid solutions. The samples were briefly sonicated for 30 s and immediately loaded onto the rheometer stage. Measurements were initiated upon mounting, using 1% strain at an angular frequency of 1 rad/s, and the modulus were recorded continuously for 1 h. Frequency sweep tests were conducted from 0.1 to 100 rad/s at 1% strain. Amplitude sweep tests were performed from 0.1% to 100% strain at a constant angular frequency of 1 rad/s. Stress relaxation tests were carried out by applying a constant strain of 10% and monitoring shear stress decay over time. Flow curves were obtained by ramping the shear rate from 0.01 to 1000 s^–1^. Interval thixotropy tests were conducted by alternating low (1%) and high (500%) strain at 1 rad/s in 1 min intervals.

### 4.6 Hydrogel Extrusion Printing

Hydrogel printing was performed using a high-precision fluid dispenser (ML-808GX; Musashi Engineering, Japan) mounted on a programmable omnidirectional direct ink writing platform (SHOT mini 200Ω×; Musashi Engineering, Japan). Printing was conducted at room temperature using a disposable syringe barrel fitted with a 21G nozzle, maintaining a fixed nozzle-to-substrate gap of 0.2 mm under pneumatic pressure control. Line extrusion performance was assessed by printing three 20 mm lines at speeds ranging from 10 to 50 mm/s. Line widths were measured by optical microscopy (n = 12). Overhang tests were conducted by printing horizontal bridge structures across progressively increasing span lengths. Multilayer grid patterns (1.25 cm × 1.25 cm, 2.5 mm spacing) were printed at 40 mm/s and incubated in PBS at 37 °C for 24 h.

### 4.7 Proteolytic Degradation

Molecular degradation was evaluated by incubating peptoid and peptide samples (0.001 wt %) with proteinase K (0.2 mg/mL) in Tris-HCl buffer (0.1 M, pH 8.0) at 37 °C. Degradation was monitored over time using MALDI-TOF mass spectrometry. Hydrogel degradation was assessed by incubating 2 wt % hydrogels (200 μL) in proteinase K solution (0.5 mg/mL, 10 mL) at 37 °C (*n* = 3). Macroscopic changes were imaged, and residual mass was measured gravimetrically. Mechanical properties were analyzed by mixing hydrogel (2 wt %, 400 μL) with proteinase K solution (0.5 mg/mL, 400 μL), followed by incubation at 37 °C and monitoring changes in storage modulus (G′) via frequency sweep rheology (*n* = 3).

### 4.8 Cargo Release Experiments

Release kinetics of FITC-dextran (4–40 kDa) from hydrogel matrices were evaluated using a dialysis-based assay (*n* = 3). FITC-dextran (0.5 mg/mL) was incorporated into the 2 wt % peptoid precursor solution immediately prior to gelation. The mixture (200 μL) was cast into syringe molds and incubated at 37 °C for 24 h. After incubation, hydrogel samples were loaded into dialysis membranes and immersed in Tris-HCl buffer (0.1 M, pH 8.0, 25 mL) at 37 °C under gentle agitation. At designated time points over 72 h, 1 mL of receptor solution was collected and replaced with fresh buffer. For proteolytic stability tests, hydrogel samples containing 4 kDa FITC-dextran were co-loaded with either buffer or proteinase K solution (0.5 mg/mL, 200 μL) into dialysis bags (*n* = 3). Membrane cutoffs were selected based on cargo size: 10 kD MWCO (SnakeSkin dialysis tubing; Thermo Fisher Scientific, USA) for 4 kDa dextran and proteinase K, and 100 kDa MWCO (SpectraPor Biotech CE dialysis tubing; Repligen, USA) for 10–40 kDa dextrans. Released FITC-dextran was quantified by PL spectroscopy (λ_ex_ = 480 nm, λ_em_ = 520 nm), and cumulative profiles were analyzed using the Higuchi diffusion model (Q = K_H_ × t^1/2^ + b_H_).

### 4.9 Cell Culture and Characterization

NIH-3T3 mouse fibroblasts and U373-MG human astrocytoma cells (Korean Cell Line Bank, Republic of Korea) were maintained in high-glucose DMEM supplemented with 10% fetal bovine serum (FBS) and 1% penicillin–streptomycin. Human adipose-derived stem cells (hADSCs, ATCC, USA) were cultured in low-glucose DMEM with 10% FBS, 1% penicillin–streptomycin, and 10 ng/mL each of bFGF and EGF. Human umbilical vein endothelial cells (HUVECs, STEMCELL Technologies, Canada) were maintained in EGM-2 medium containing 2% FBS and growth supplements. Rat cortical neurons (E18 Sprague–Dawley rats; Young Bio, Republic of Korea) were isolated following protocols approved by the Institutional Animal Care and Use Committee of the Korea Institute of Science and Technology (KIST-2022-071). Neurons were enzymatically dissociated using papain (Miltenyi Biotec, Germany), plated on poly-d-lysine–coated surfaces (PDL, Thermo Fisher Scientific, USA), and cultured in Neurobasal medium supplemented with B-27, GlutaMAX, and 1% penicillin–streptomycin. Unless otherwise noted, all media and reagents were purchased from Thermo Fisher Scientific (USA).

NIH-3T3 fibroblasts (5.00 × 10^4^ cells/well) were seeded onto preformed 2 wt % *Noct*(*Npm*)_4_GRGD hydrogels mounted in 4-well plates after PBS preincubation and cultured for up to 4 days under standard conditions (*n* = 4). At designated time points, cell viability and proliferation were assessed via LIVE/DEAD staining and quantified with ImageJ. For cytotoxicity assays, rat cortical neurons, U373-MG, hADSCs, NIH-3T3, and HUVECs (8.68 × 10^3^ cells/well) were seeded into 96-well plates and treated with *Noct*(*Npm*)_4_GRGD solution (0–100 μg/mL) for 24 h (*n* = 4). PDL-coated wells served as controls. Viability was evaluated using a LIVE/DEAD assay. The data are presented as mean ± SD from four independent experiments (*n* = 4). To assess nanostructure uptake, NIH-3T3 cells were incubated with *Noct*(*Npm*)_4_GRGD or (*Npm*)_4_GRGD (50 μM, 5% FAM-labeled) in serum-free medium for 24 h. Following incubation, cells were gently washed three times with PBS to remove noninternalized materials prior to fixation. After fixation, cells were stained with phalloidin rhodamine and DAPI, and imaged by confocal microscopy (LM700; Zeiss, Germany). For cargo delivery experiments, FITC-dextran (0.5 mg/mL) was embedded in 2 wt % *Noct*(*Npm*)_4_GRGD hydrogels and preincubated in PBS. NIH-3T3 fibroblasts (5.00 × 10^4^ cells/well) were then seeded on the gels and cultured for 24 hours (*n* = 4). Following fixation, intracellular FITC fluorescence was visualized by confocal microscopy and quantified using ImageJ from at least five random fields (*n* = 3).

### 4.10 Statistical Analysis and Reproducibility

All data are presented as mean ± SD from at least three independent experiments, unless otherwise specified.

## Supplementary Material





## Notes

HPLC/UPLC and MALDI-TOF analyses of peptoid hydrogelators, nanostructure characterization (DLS, TEM, cryo-TEM, AFM, WAXS), optical properties (UV–Vis, photoluminescence), hydrogelation and morphology, molecular modeling, proteolytic stability with proteinase K, cargo release and diffusion analysis, and cellular uptake of FITC-dextran cargos (Figures S1–S17) (PDF)
Time-lapse observation of peptoid self-assembly dynamics (Movie S1) (MP4)
