# Supplementary material for: N‑Terminal Octylated Peptoid Hydrogels as 3D-Printable Cell Scaffolds and Proteolytically Robust Cargo Depots
Source: ACS Nano. 2026 Feb 9;20(7):5650–61. doi: 10.1021/acsnano.5c16998 (PMC12947725; doi:10.1021/acsnano.5c16998)
Supplement: Supplementary file 1 [file nn5c16998_si_001.pdf]

Supporting Information

N-terminal Octylated Peptoid Hydrogels as 3D-Printable Cell Scaffolds and Proteolytically Robust Cargo Depots

Il-Soo Park[†], *Younghak Cho*[‡], *Yen Jea Lee*[§], *Daniela Gutierrez*[¶], *Ronald N. Zuckermann*[‖], *Hyejeong Seong*[‡#*], *Jae Hong Kim*[†⊥ȼ*]

† Electronic and Hybrid Materials Research Center, Korea Institute of Science and Technology, Seoul 02792, Republic of Korea

‡ Brain Science Institute, Korea Institute of Science and Technology, Seoul 02792, Republic of Korea

§ Materials Sciences Division, Lawrence Berkeley National Laboratory, California 94720, United States

¶ Biomaterials Research Center, Biomedical Research Division, Korea Institute of Science and Technology, Seoul 02792, Republic of Korea

‖ Molecular Foundry, Lawrence Berkeley National Laboratory, California 94720, United States

Division of Bio-Medical Science and Technology, KIST School, University of Science and Technology, Seoul 02792, Republic of Korea

⊥ Convergence Research Center for Solutions to Electromagnetic Interference in Future-mobility, Korea Institute of Science and Technology, Seoul 02792, Republic of Korea

ȼ GIST InnoCORE AI-Nano Convergence Institute for Early Detection of Neurodegenerative Diseases, Gwangju Institute of Science and Technology, Gwangju 61005, Republic of Korea

*These authors are co-corresponding authors.
*E-mail: jaehongkim@kist.re.kr; h.seong@kist.re.kr

Keywords: Peptoid, Hydrogels, Self-assembly, Cell Scaffolds, Protease-resistant materials, Cargo delivery

Table of contents

Figure S1. Inverted vial image of 1 wt% (*Npm*)$_4$GRGD in PBS buffer after 24 hours of incubation at room temperature 3

Figure S2. UPLC chromatograms and MALDI-TOF mass spectra of *Noct*(*Npm*)$_4$GRGD and (*Npm*)$_4$GRGD peptoid hydrogelator 4

Figure S3. Derived count rate of *Noct*(*Npm*)$_4$GRGD and (*Npm*)$_4$GRGD at varying concentrations, measured by DLS 5

Figure S4. Cryo-TEM image of *Noct*(*Npm*)$_4$GRGD at 1 wt% in aqueous solution 6

Figure S5. TEM image of (*Npm*)$_4$GRGD at 1 wt% in aqueous solution 7

Figure S6. WAXS profiles of *Noct*(*Npm*)$_4$GRGD and (*Npm*)$_4$GRGD at 1 wt% in aqueous solution 8

Figure S7. AFM image of *Noct*(*Npm*)$_4$GRGD at 1 wt% in aqueous solution 9

Figure S8. UV–Vis absorption spectra of *Noct*(*Npm*)$_4$GRGD and (*Npm*)$_4$GRGD at 1 wt% in water and a 50:50 (v/v) acetonitrile (ACN)/water mixture 10

Figure S9. PL spectra of *Noct*(*Npm*)$_4$GRGD and (*Npm*)$_4$GRGD in aqueous solution at varying concentrations (0.015 – 1 wt%), and plot of the emission intensity ratio (I_{287}/I_{281}) as a function of concentration 11

Figure S10. Molecular structure of *Noct*(*Npm*)$_4$GRGD visualized using PyMOL 12

Figure S11. Time-sweep rheology of *Noct*(*Npm*)$_4$GRGD hydrogelation 16

Figure S12. Temperature-dependent gelation behavior and mechanical stability of 2 wt% *Noct*(*Npm*)$_4$GRGD hydrogel 17

Figure S13. Inverted vial images of 2 wt% *Noct*(*Npm*)$_4$GRGD hydrogels prepared at pH 5–8 12

Figure S14. MALDI-TOF mass spectra of *Noct*(*Npm*)$_4$GRGD and Fmoc-FF after proteinase K treatment 16

Figure S15. Cumulative release profiles of *Noct*(*Npm*)$_4$GRGD and Fmoc-FF hydrogels, with or without proteinase K, fitted to the Higuchi diffusion model 17

Figure S16. Fluorescence microscopy images showing cellular uptake of FITC-Dextran cargos (4, 10, 20, and 40 kDa) by NIH-3T3 fibroblast cells 18

Figure S17. Chemical structures, HPLC chromatograms and MALDI-TOF mass spectra of FAM-labeled peptoid hydrogelators 19

Movie S1. Time-lapse visual observation of spontaneous self-assembly in 1 wt% *Noct*(*Npm*)$_4$GRGD aqueous solution

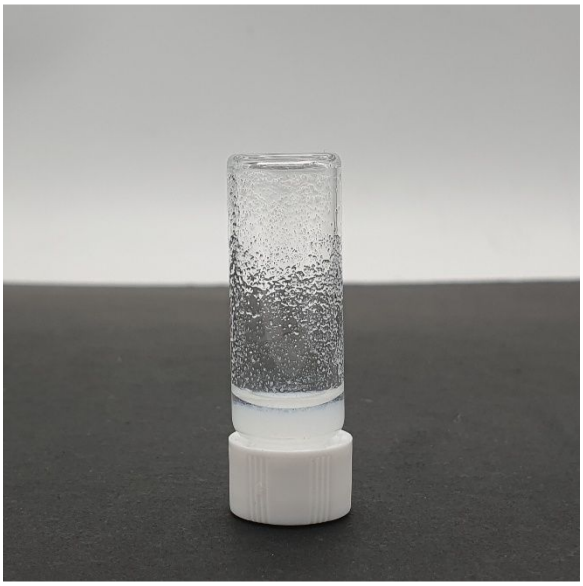

Figure S1. Inverted vial image of 1 wt% (*Npm*)$_4$GRGD in PBS buffer after 24 hours of incubation at room temperature. The solution exhibits precipitation and fails to undergo gelation.

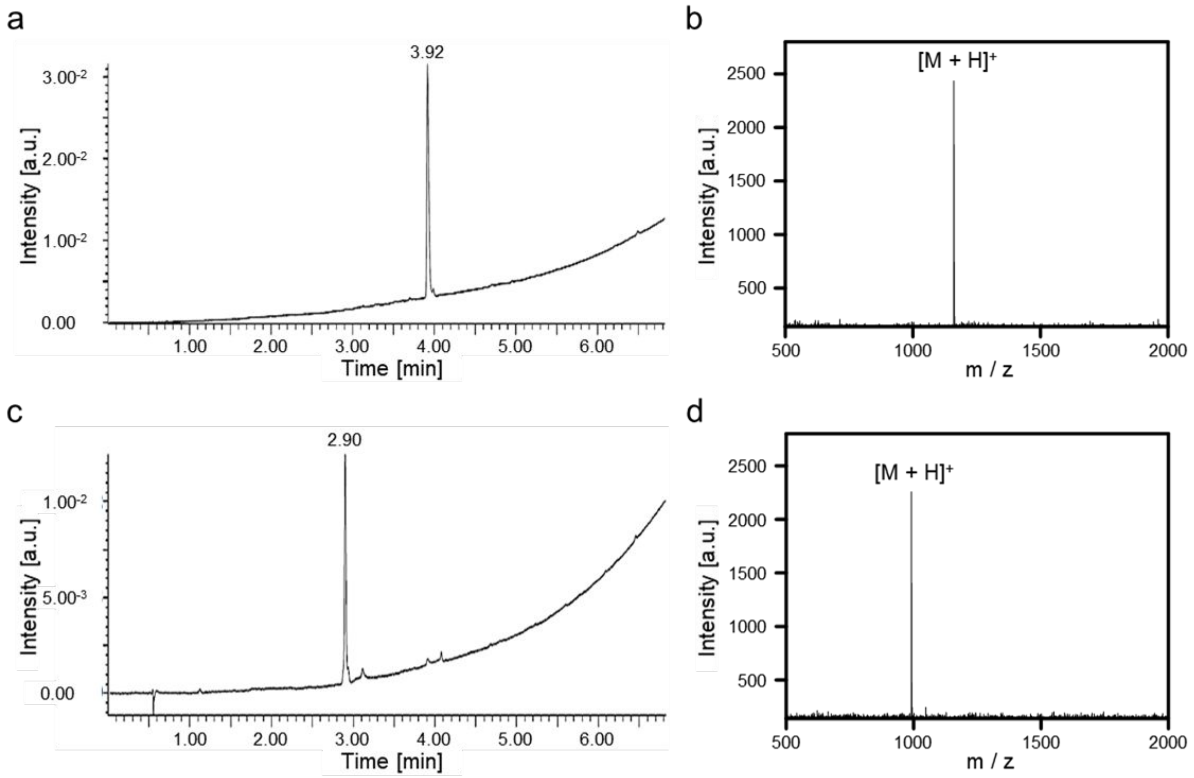

Figure S2. (a) UPLC chromatogram and (b) MALDI-TOF mass spectrum of *Noct*(*Npm*)$_4$GRGD peptoid hydrogelator. The observed mass peak at m/z 1160.382 corresponds to the $[M+H]^+$ peak (calcd. 1160.62). (c) UPLC chromatogram and (d) MALDI-TOF mass spectrum of (*Npm*)$_4$GRGD peptoid hydrogelator. The observed mass peak at m/z 991.242 corresponds to the $[M+H]^+$ peak (calcd. 991.47). UPLC was performed using a linear gradient of 5 – 95% acetonitrile (0.1% TFA) over 6.8 min at a flow rate of 0.6 mL/min and a column temperature of 60 °C. UV/visible detector was set to monitor sample elution at 254 nm.

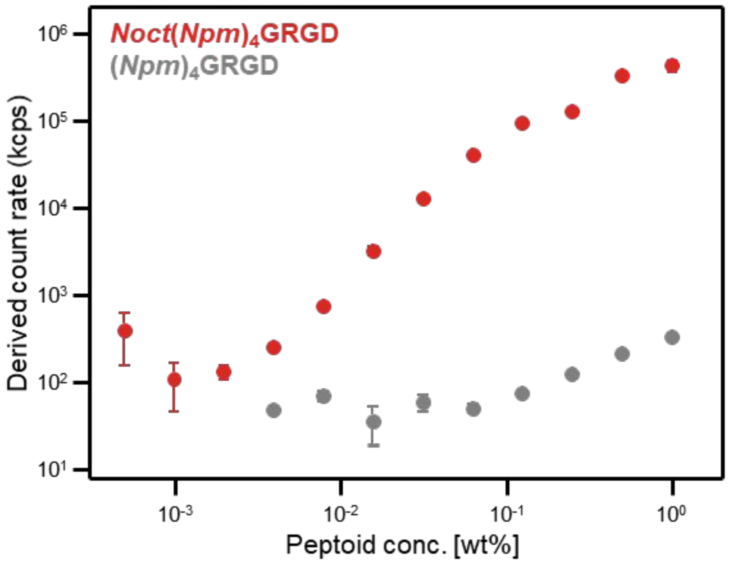

Figure S3. Derived count rate of *Noct*(*Npm*)$_4$GRGD and (*Npm*)$_4$GRGD at varying concentrations, measured by DLS (n=3).

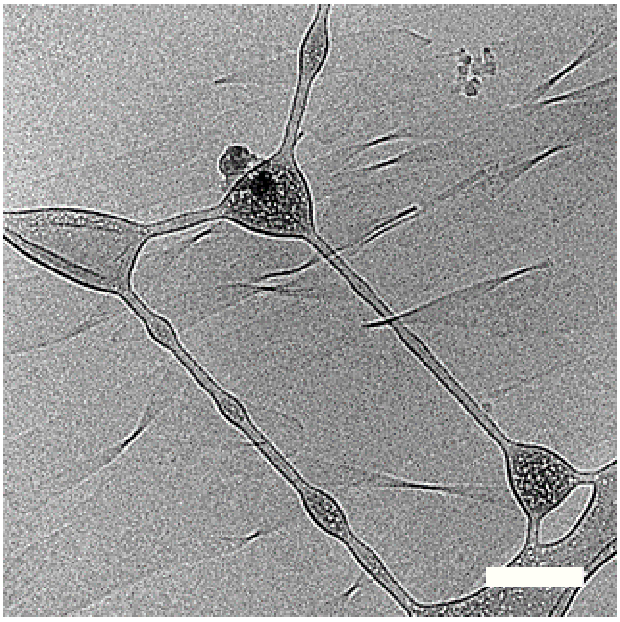

Figure S4. Cryo-TEM image of *Noct*(*Npm*)$_4$GRGD at 1 wt% in aqueous solution. The scale bar represents 200 nm.

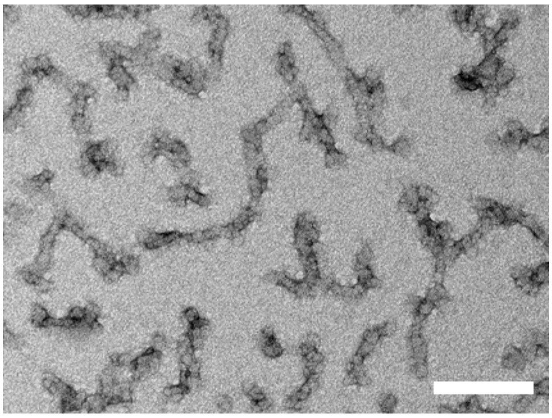

Figure S5. TEM image of (Npm)$_4$GRGD at 1 wt% in aqueous solution. The scale bar represents 200 nm.

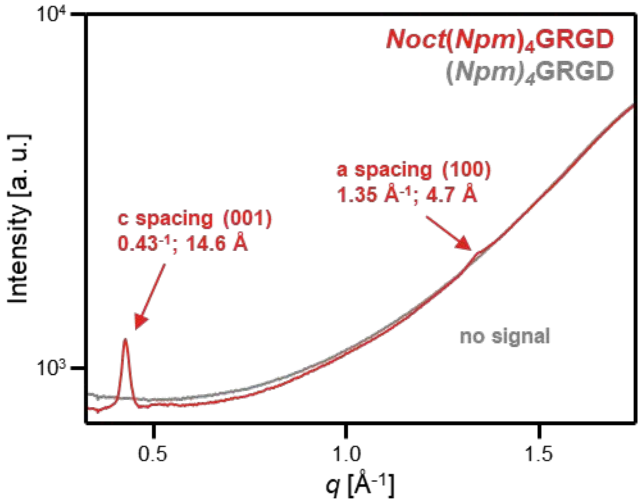

Figure S6. WAXS profiles of *Noct*(*Npm*)$_4$GRGD (red) and (*Npm*)$_4$GRGD (gray) at 1 wt% in aqueous solution. No ordered signal was observed for (*Npm*)$_4$GRGD.

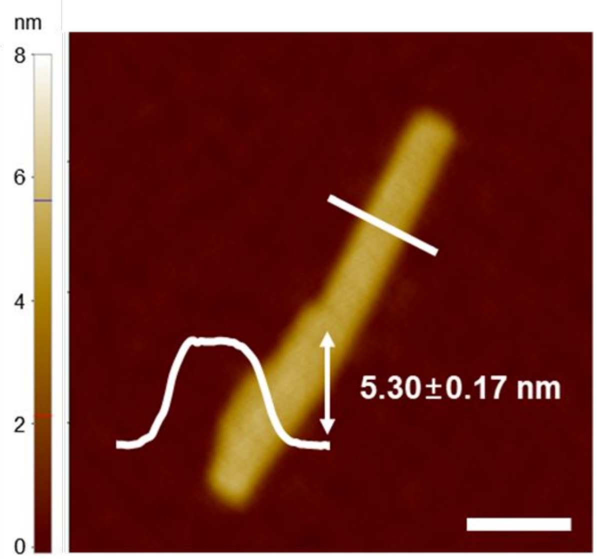

Figure S7. AFM image of *Noct*(*Npm*)$_4$GRGD at 1 wt% in aqueous solution. The corresponding height profile indicates a nanosheet thickness of approximately 5.30 ± 0.17 nm (n=10). The scale bar represents 200 nm.

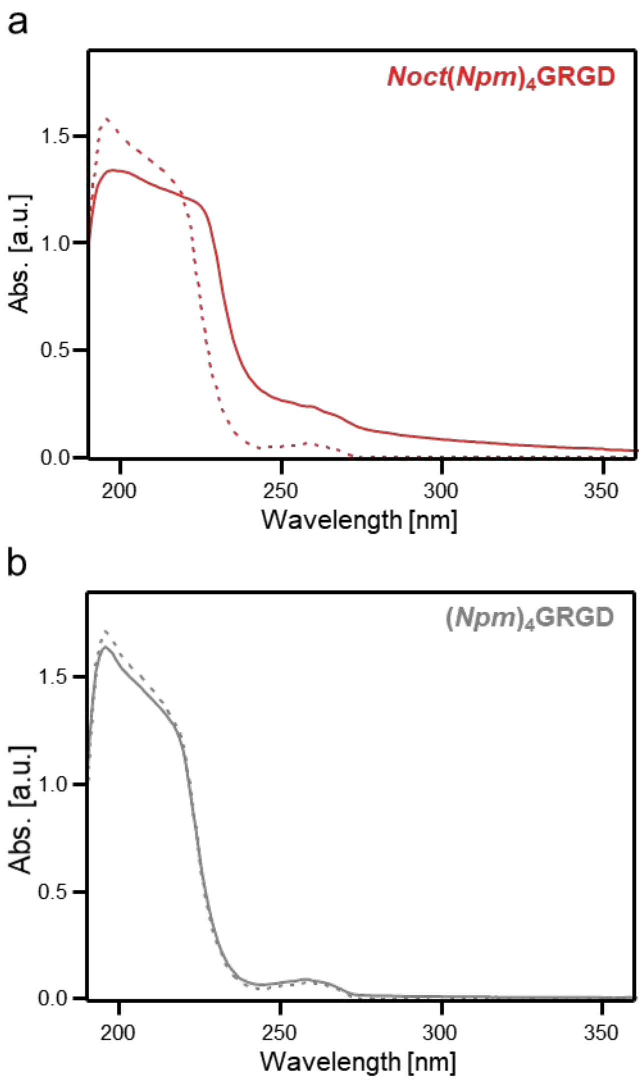

Figure S8. UV–Vis absorption spectra of (a) *Noct*(*Npm*)$_4$GRGD and (b) (*Npm*)$_4$GRGD at 1 wt% in water (solid line) and a 50:50 (v/v) acetonitrile (ACN)/water mixture (dashed line).

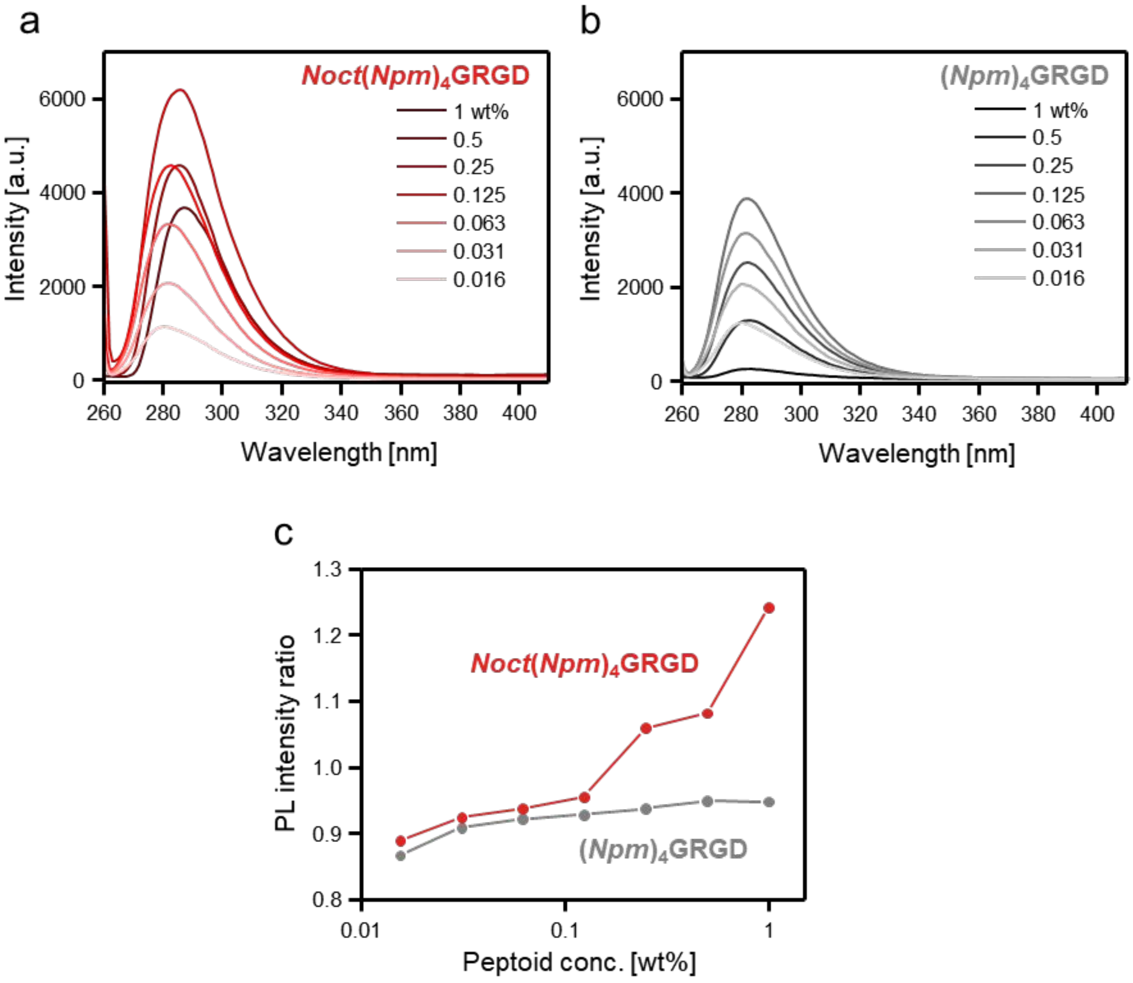

Figure S9. PL spectra of (a) *Noct*(*Npm*)$_4$GRGD and (b) (*Npm*)$_4$GRGD in aqueous solution at varying concentrations (0.015 – 1 wt%). (c) Plot of the emission intensity ratio (I_{287}/I_{281}) as a function of concentration, corresponding to the peaks at 287 nm and 281 nm.

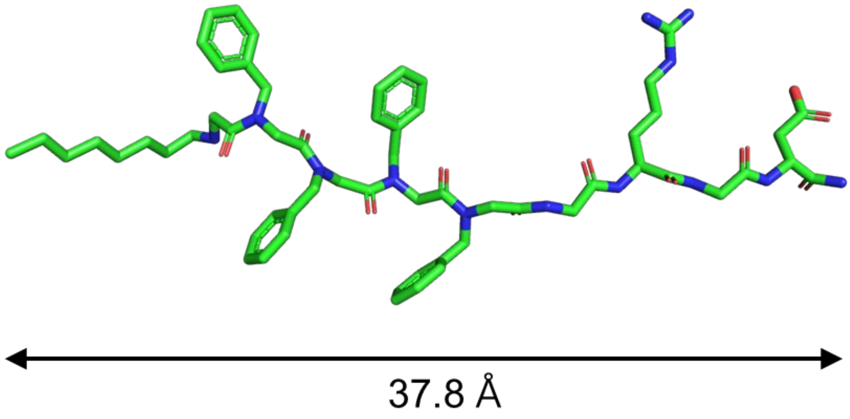

Figure S10. Molecular structure of *Noct*(*Npm*)$_4$GRGD visualized using PyMOL. The overall molecular length is approximately 37.8 Å.

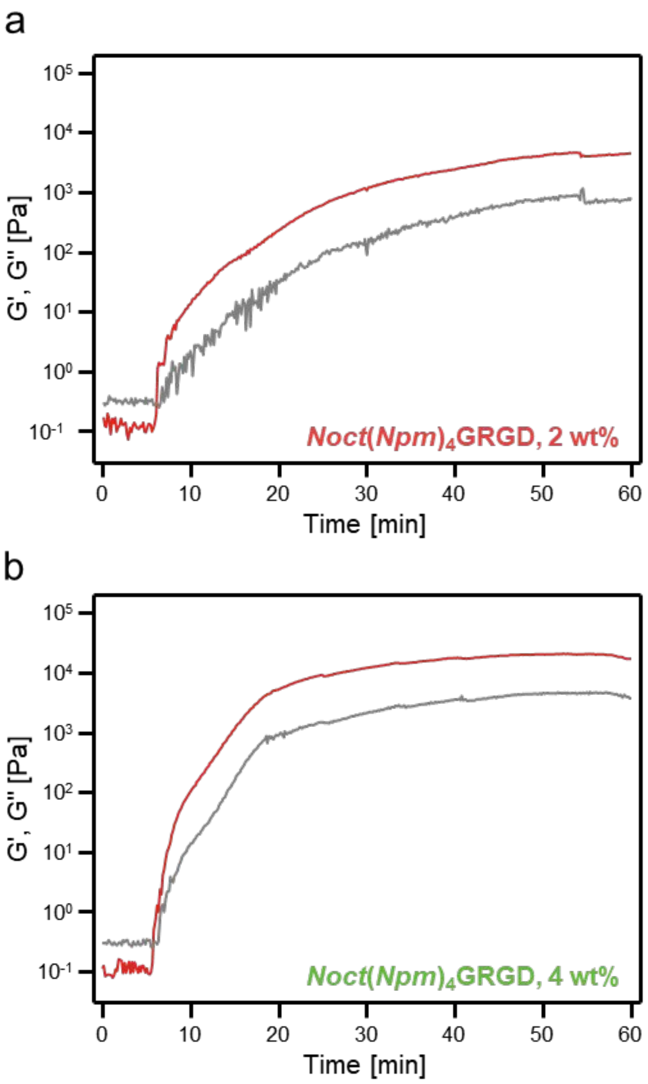

Figure S11. Time-sweep rheology of *Noct*(*Npm*)$_4$GRGD hydrogelation at (a) 2 wt% and (b) 4 wt%. Storage modulus (G', red) and loss modulus (G'', gray) were monitored over time.

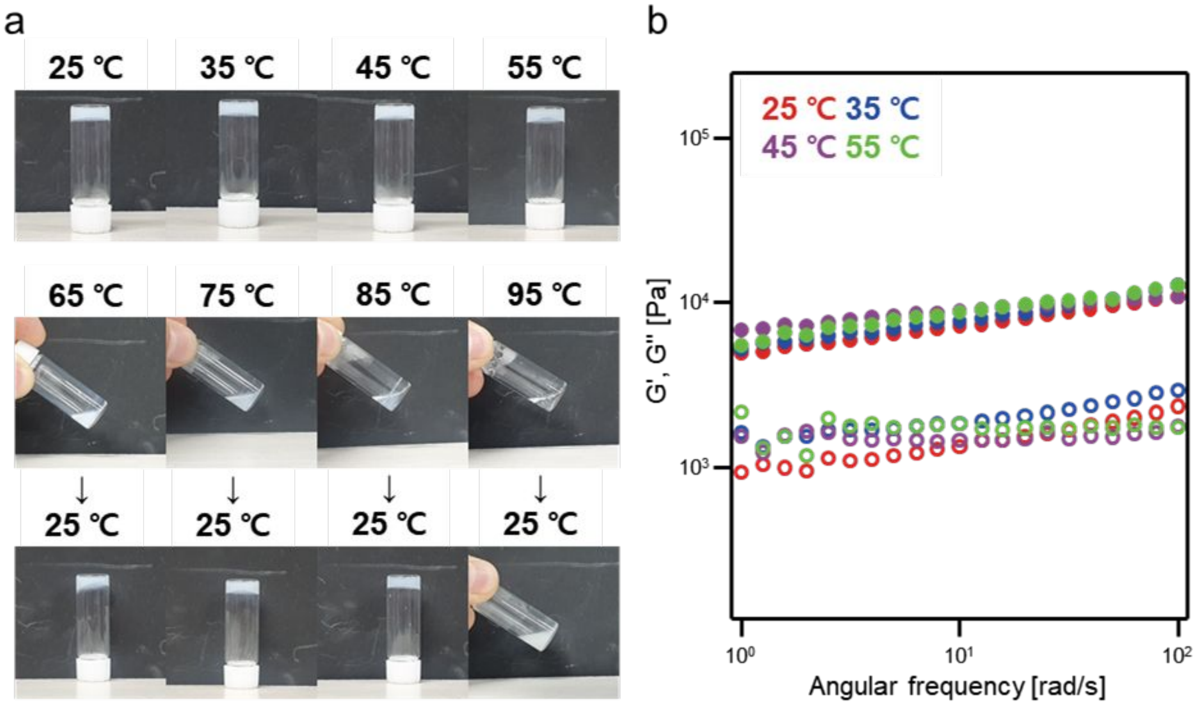

Figure S12. Temperature-dependent gelation behavior and mechanical stability of 2 wt% *Noct*(*Npm*)$_4$GRGD hydrogel. (a) Inverted-vial images of *Noct*(*Npm*)$_4$GRGD hydrogel measured from 25 to 95 °C Samples were incubated at each temperature for 10 min before assessment. The gel state was maintained up to 55 °C, whereas samples transitioned to a sol state above 65 °C. Upon cooling to 25 °C, samples heated up to 85 °C recovered their gel-like behavior, while those heated to 95 °C formed a transparent sol that became turbid upon cooling but did not regain gel characteristics. (b) Frequency-sweep rheology of 2 wt% *Noct*(*Npm*)$_4$GRGD hydrogels measured in the gel state (25–55 °C). Both G' (filled circle) and G'' (open circle) exhibit minimal temperature-dependent variation.

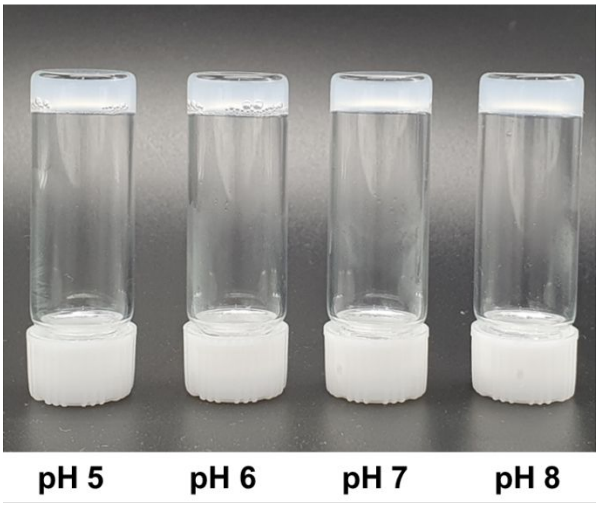

Figure S13. Inverted-vial images of 2 wt% *Noct*(*Npm*)$_4$GRGD hydrogels prepared at pH 5–8.

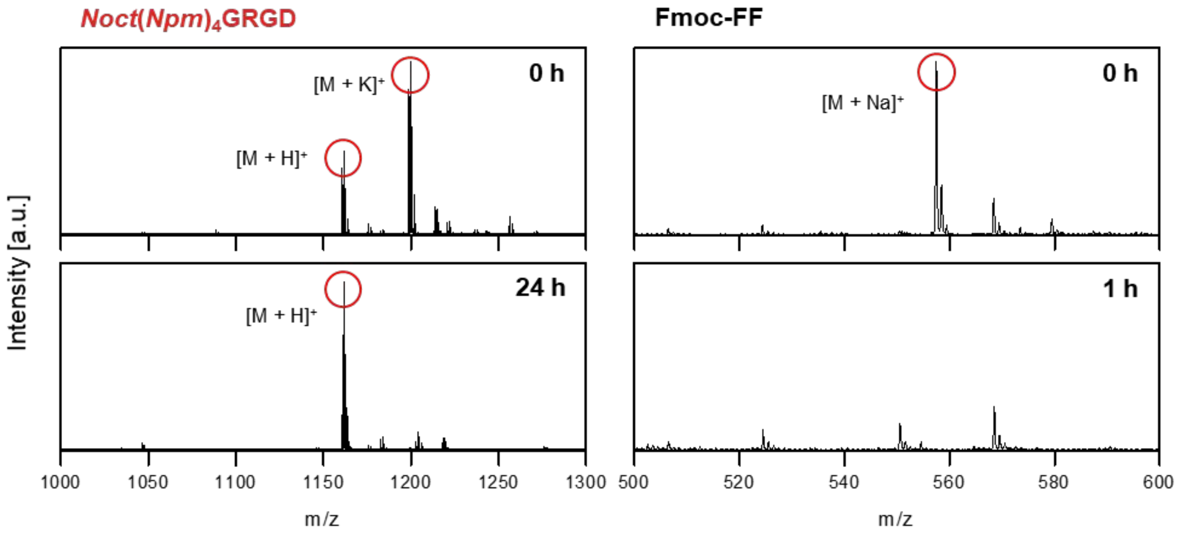

Figure S14. MALDI-TOF mass spectra of *Noct*(*Npm*)$_4$GRGD and Fmoc-FF after proteinase K treatment. Samples were prepared at 0.001 wt% and incubated with 0.2 mg/mL proteinase K for 1 hour (Fmoc-FF) or 24 hours (*Noct*(*Npm*)$_4$GRGD). For *Noct*(*Npm*)$_4$GRGD ($C_{60}H_{81}N_{13}O_{11}$), the $[M+H]^+$ peak was detected at m/z 1161.78 (calcd. 1160.62) and the $[M+K]^+$ peak at 1199.79 (calcd. 1198.62). For Fmoc-FF ($C_{33}H_{30}N_2O_5$), the $[M+Na]^+$ peak was detected at m/z 557.44 (calcd. 557.22).

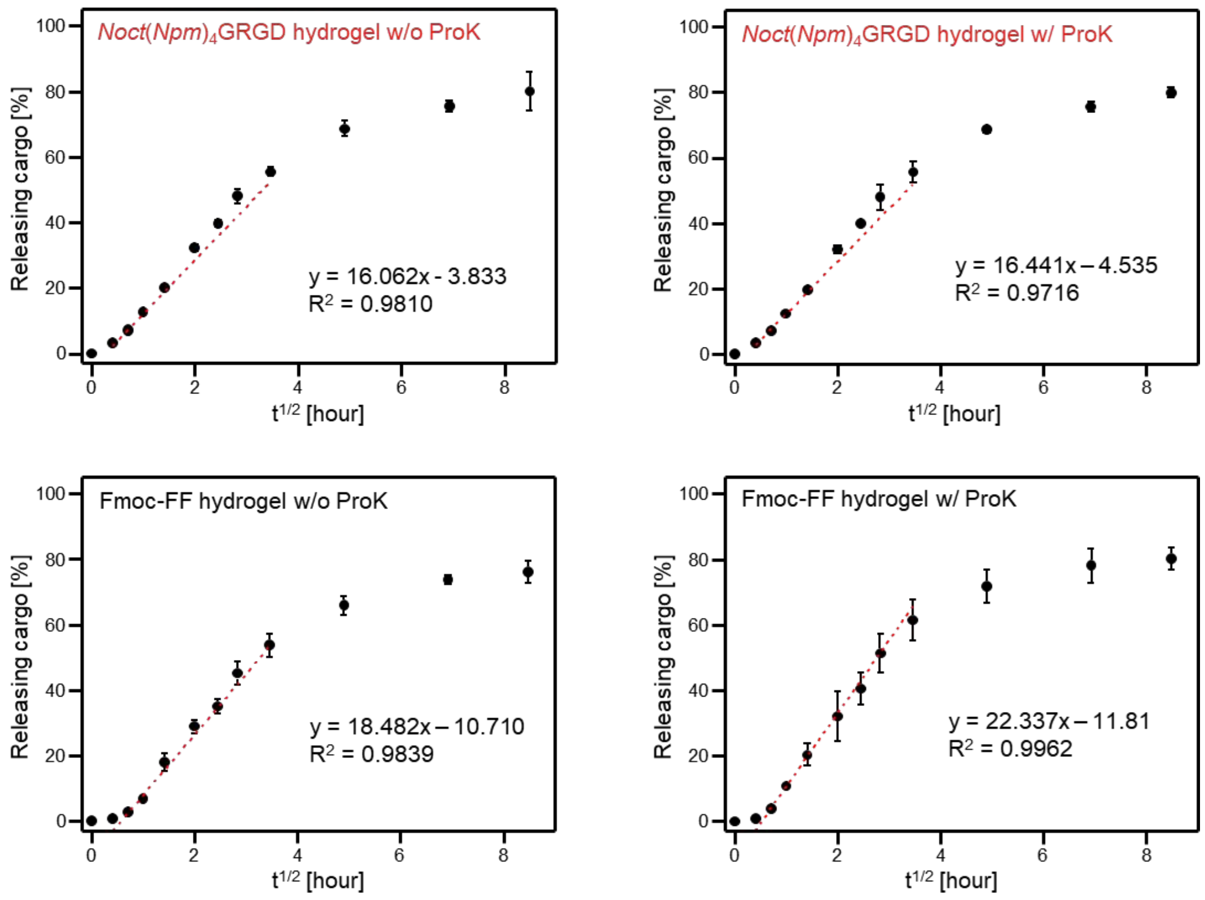

Figure S15. Cumulative release profiles of *Noct*(*Npm*)$_4$GRGD and Fmoc-FF hydrogels, with or without proteinase K (n=3), were fitted to the Higuchi diffusion model. To maintain the validity of the model, only the initial linear region was considered for fitting, and data points deviating from linearity at extended time points were excluded. The fitted curves exhibited small negative intercepts, likely reflecting minor experimental variation during initial sampling.

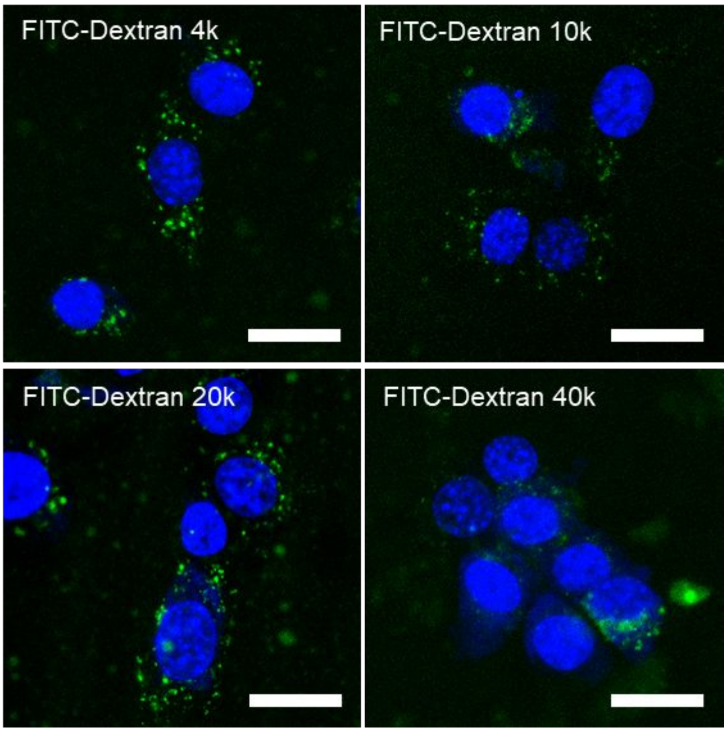

Figure S16. Fluorescence microscopy images showing cellular uptake of FITC-Dextran cargos (4, 10, 20, and 40 kDa) by NIH-3T3 fibroblast cells. Cells were seeded onto *Noct*(*Npm*)$_4$GRGD hydrogels embedded with FITC-Dextran (green) and incubated for 24 hours. The scale bar represents 20 µm.

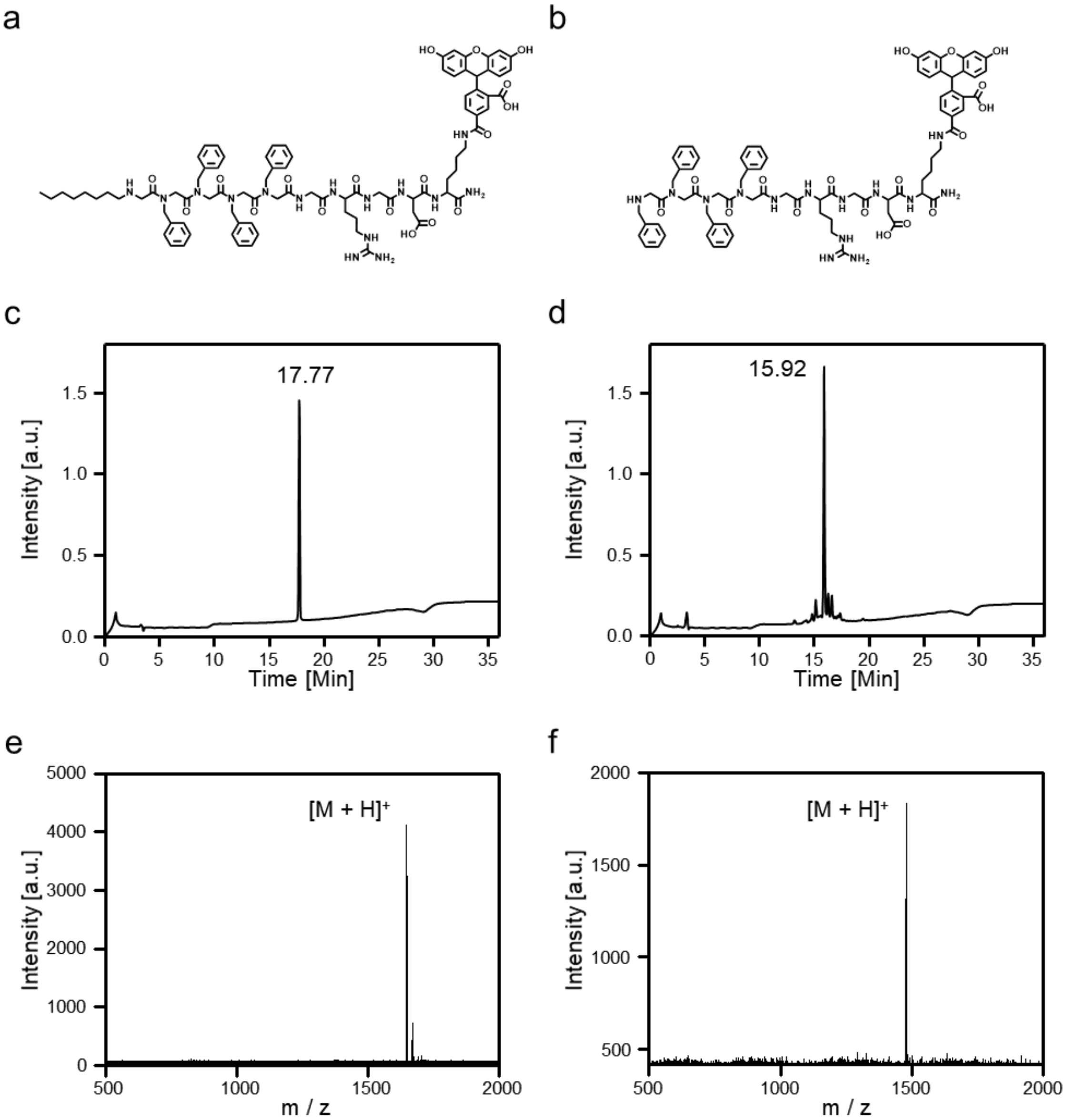

Figure S17. Chemical structures of FAM-labeled peptoid hydrogelators, (a) *Noct*(*Npm*)$_4$GRGDK(FAM) and (b) (*Npm*)$_4$GRGDK(FAM). (c) HPLC chromatogram and (e) MALDI-TOF mass spectrum of *Noct*(*Npm*)$_4$GRGDK(FAM) peptoid hydrogelator. The observed mass peak at m/z 1646.603 corresponds to the $[M+H]^+$ peak (calcd. 1648.78). (d) HPLC chromatogram and (f) MALDI-TOF mass spectrum of (*Npm*)$_4$GRGDK(FAM) peptoid hydrogelator. The observed mass peak at m/z 1477.398 corresponds to the $[M+H]^+$ peak (calcd. 1479.63). HPLC was performed at a flow rate of 1.0 mL/min with a linear gradient of 10 – 100% acetonitrile (0.1% TFA) over 20 min, following a 5 min isocratic hold at 10% acetonitrile after sample injection. UV/visible detector was set to monitor sample elution at 220 nm.
